# ATP-dependent G-quadruplex unfolding by Bloom helicase exhibits low processivity

**DOI:** 10.1093/nar/gkv531

**Published:** 2015-05-18

**Authors:** Jagat B. Budhathoki, Edward J. Stafford, Jaya G. Yodh, Hamza Balci

**Affiliations:** 1 Department of Physics, Kent State University, Kent, OH 44242, USA; 2 Department of Physics and Center for the Physics of Living Cells, University of Illinois at Urbana-Champaign, Urbana, IL 61801, USA

## Abstract

Various helicases and single stranded DNA (ssDNA) binding proteins unfold G-quadruplex (GQ) structures. However, the underlying mechanisms of this activity have only recently come to focus. We report kinetic studies on Bloom (BLM) helicase and human telomeric GQ interactions using single-molecule Förster resonance energy transfer (smFRET). Using partial duplex DNA (pdDNA) constructs with different 5′ ssDNA overhangs, we show that BLM localizes in the vicinity of ssDNA/double-stranded DNA (dsDNA) junction and reels in the ssDNA overhang in an ATP-dependent manner. A comparison of DNA constructs with or without GQ in the overhang shows that GQ unfolding is achieved in 50–70% of reeling attempts under physiological salt and pH conditions. The unsuccessful attempts often result in dissociation of BLM from DNA which slows down the overall BLM activity. BLM-mediated GQ unfolding is typically followed by refolding of the GQ, a pattern that is repeated several times before BLM dissociates from DNA. BLM is significantly less processive compared to the highly efficient GQ destabilizer Pif1 that can repeat GQ unfolding activity hundreds of times before dissociating from DNA. Despite the variations in processivity, our studies point to possible common patterns used by different helicases in minimizing the duration of stable GQ formation.

## INTRODUCTION

Human Bloom helicase (BLM) is classified as a superfamily 2 helicase (1,2) . More specifically BLM is a member of RecQ family of helicases and contains two RecA-like domains that are involved in binding and hydrolysis of ATP and enable BLM to translocate on ssDNA in the 3′ to 5′ direction (2–5). In addition to its dsDNA unwinding activity, BLM is known to resolve non-canonical DNA structures (6–9), such as intermolecular and intramolecular G-quadruplex (GQ) structures (10–14). Deficiencies in BLM give rise to Bloom syndrome, characteristics of which include genomic instability, increased predisposition to cancer, infertility and dwarfism (15,16). In particular, loss of BLM helicase has been shown to result in increased DNA breaks and genomic instability in potential GQ-forming sites of the genome (6).

Bulk biophysical studies have demonstrated that BLM unfolds both intermolecular and intramolecular GQ structures in the presence of ATP (8,9,17–19), while recent single-molecule studies demonstrated that BLM destabilizes GQ in the absence of ATP as well (20,21). hWRN helicase also shows GQ unfolding in the absence of ATP; however, human RECQ5 and *Escherichia coli* RecQ are significantly less efficient in GQ unfolding under similar conditions (20). We have shown that the efficiency of BLM-mediated GQ unfolding activity was enhanced in the presence of non-hydrolyzable ATP analogs, ATPγS and AMP-PNP, and reduced in the presence of the hydrolysis product, ADP, which correlates with the effect these nucleotides have on BLM-ssDNA binding stability. A similar correlation between DNA-binding stability and protein-mediated GQ unfolding was also observed in ssDNA-binding proteins such as Replication Protein A (RPA) and Protection of Telomere 1 (POT1) (22,23). This similarity suggested a common initiating mechanism for GQ destabilization in the absence of ATP for ssDNA-binding proteins and enzymes that need to bind to the vicinity of GQ before unfolding it. For both groups of proteins an ssDNA overhang in the vicinity of GQ is required for GQ unfolding, and whether this overhang is placed 3′ or 5′ to the GQ made a significant difference depending on the polarity of the protein orientation (20).

In the current study, we have examined BLM-mediated GQ unfolding activity in the presence of ATP using smFRET. The 5′ ssDNA overhang of the pdDNA constructs contained either a polythymine (poly-T) sequence or the human telomeric GQ-forming sequence and a poly-T spacer between the duplex region and GQ, to accommodate BLM binding in the vicinity of the ssDNA/dsDNA junction. Under these conditions, we observed that BLM localizes in the vicinity of the junction and reels in the ssDNA tail before encountering the GQ. Upon encountering the GQ, BLM unfolds the GQ in 50–70% of the cases, and dissociates from DNA without unfolding the GQ in the other cases. The reeling activity is also observed for poly-T ssDNA overhangs which do not contain a GQ. A comparison of these results with those on Pif1 helicase (24) suggests processivity is a significant factor in the efficiency of helicase-mediated GQ destabilization.

## MATERIALS AND METHODS

### DNA and protein constructs

We utilized smFRET assay to examine the activity of BLM^642–1290^ on partial duplex DNA substrates under physiologically relevant salt (150 mM K^+^, 5 mM Mg^2+^) and pH (7.5) conditions. BLM^642–1290^ comprises the RecQ core of BLM containing the helicase, RecQ C-terminal (RQC) domain and helicase and RNase D C-terminal (HRDC) domain, and it maintains both dsDNA unwinding and ssDNA translocation activities of wild-type protein (25). Two types of DNA constructs were used in this study: those that include a GQ-forming sequence in the ssDNA overhang (pd-12ThGQ, pd-30ThGQ, pd-30ThGQ-12bp and pd-28TCy3hGQ) and those that are comprised of only poly-T sequence in the overhang (pd-35T and pd-50T). The schematics of all DNA constructs are shown in respective figures along with the data on these constructs, and sequences are given in Supplementary Table S1. The polarity with a 5′ free-end of partial duplex DNA constructs is selected to avoid unwinding of the duplex stem by BLM, as BLM would translocate away from the duplex when ATP is present. pd-35T has the same overhang length as that of pd-12ThGQ (both 35 nt) and pd-50T has a similar overhang length as that of pd-30ThGQ (50 versus 53 nt). The overhangs of pd-35T and pd-50T do not form any secondary structure, and in this respect they serve as reference structures for the GQ-forming constructs. pd-12ThGQ and pd-30ThGQ have 12T and 30T spacers, respectively, between the duplex and quadruplex to enable binding of BLM to the spacer site and its interaction with the GQ. pd-30ThGQ-12bp has a 30T spacer on the 3′ side of GQ and a 12 bp duplex on the 5′ side. pd-28TCy3hGQ has a 30T spacer but is internally Cy3-labeled at the 28th thymine from the junction.

### Experimental method and analysis of DNA reeling events

Prism-type total internal reflection microscopy was used to perform the measurements. Details of the assay conditions are given in Supplementary Data. The total number of observed events in several hundred traces was counted and this number was divided by the total time, which yields the Event Rate (s^−1^). Traces that did not show any reeling events were also included in the statistics in order to provide a better representation of the concentration dependence.

## RESULTS

### BLM-mediated GQ unfolding in the presence of ATP

**Figure 1.**
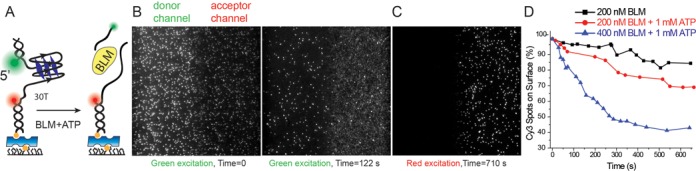
(**A**) Schematic of DNA construct used in this study and a depiction of the decrease in the number of surface bound donor fluorophore (Cy3, green) as the short duplex is unwound. The acceptor fluorophore (Cy5, red) remains bound to surface even after the short duplex is unwound as it is attached with a biotin-neutravidin linker. (**B**–**D**) Duplex unwinding, as monitored by counting Cy3 spots that remain on the surface, is used as a proxy for GQ unfolding in pd-30ThGQ12bp construct. (B) Image of a section of the sample at time *t* = 0 s and *t* = 122 s as obtained via green laser (*λ* = 532 nm) excitation. (C) Image of the same section as in (B) at time *t* = 710 s as obtained via red laser (*λ* = 633 nm) excitation. (D) Plot of fraction of remaining Cy3 spots as a function of time for 200 nM BLM (black), 200 nM BLM + 1 mM ATP (red) and 400 nM BLM + 1 mM ATP (blue). The fraction of remaining Cy3 spots reduces faster as BLM and ATP concentrations are increased. Note, the data in the absence of ATP are essentially a measure of Cy3 photobleaching.

After characterizing ATP-independent BLM-mediated GQ unfolding (14), we next sought to examine the impact of ATP on this activity. First, in order to conclusively demonstrate GQ unfolding by BLM, we designed the construct (pd-30ThGQ12bp) shown in Figure 1A. The only site to which BLM can initially bind in this construct is the 30T spacer on the 3′-side of GQ. Therefore, we predict that BLM will only be able to unwind the 12 bp duplex if it first unfolds GQ.

Unwinding of the 12 bp duplex results in release of donor fluorophore, thus providing a means to detect GQ unfolding (see Figure 1A for a schematic of this measurement). To quantify duplex unwinding, we measured the number of Cy3 spots remaining within the imaging area as a function of time after addition of BLM and ATP at 150 mM K^+^ (pH 7.5). Figure 1B (left panel) shows an initial image of the donor and acceptor channels under 532 nm green laser illumination before BLM and ATP are added (*t* = 0 s). Figure 1B (right panel) shows a significant decrease in the number of Cy3 spots within the same area with green laser excitation 122 s after addition of 400 nM BLM + 1 mM ATP. As expected, red laser excitation of the same region (Figure 1C) shows the majority of acceptor spots remain attached to the surface via a biotin-neutravidin linker (measured 710 s after addition of BLM and ATP). Quantification of the time-dependence of loss of Cy3 spots for different BLM and ATP concentrations (Figure 1D) confirms that Cy3 spots are removed faster at higher BLM concentrations. These results suggest that BLM is capable of unfolding the GQ as evidenced by unwinding of the 12 bp duplex DNA in this construct.

It is conceivable that BLM might somehow bind to GQ or make contact with the short spacer between the duplex and GQ and progress to unwinding the duplex without unfolding the GQ. To address this possibility, we designed a control measurement in which a duplex DNA is placed on the 5′ side of a GQ that has a short enough overhang that BLM should not be able to bind stably enough to unfold it (Supplementary Figure S1). However, a stable GQ and a short spacer (2 nt) are still maintained in this construct and BLM should still be able to make contact with these regions. These measurements did not show any duplex unwinding under these circumstances. In addition, as the GQ overhang was only 4 nt long, BLM could not initiate GQ unfolding by binding to this region, which resulted in all GQ molecules remaining stably folded at even the highest BLM and ATP concentrations we studied. In conclusion, these measurements show that BLM does not bypass a folded GQ and proceed to unwind the duplex by either making contact with the GQ itself without unfolding it, or with the short spacer between the GQ and the duplex.

**Figure 2.**
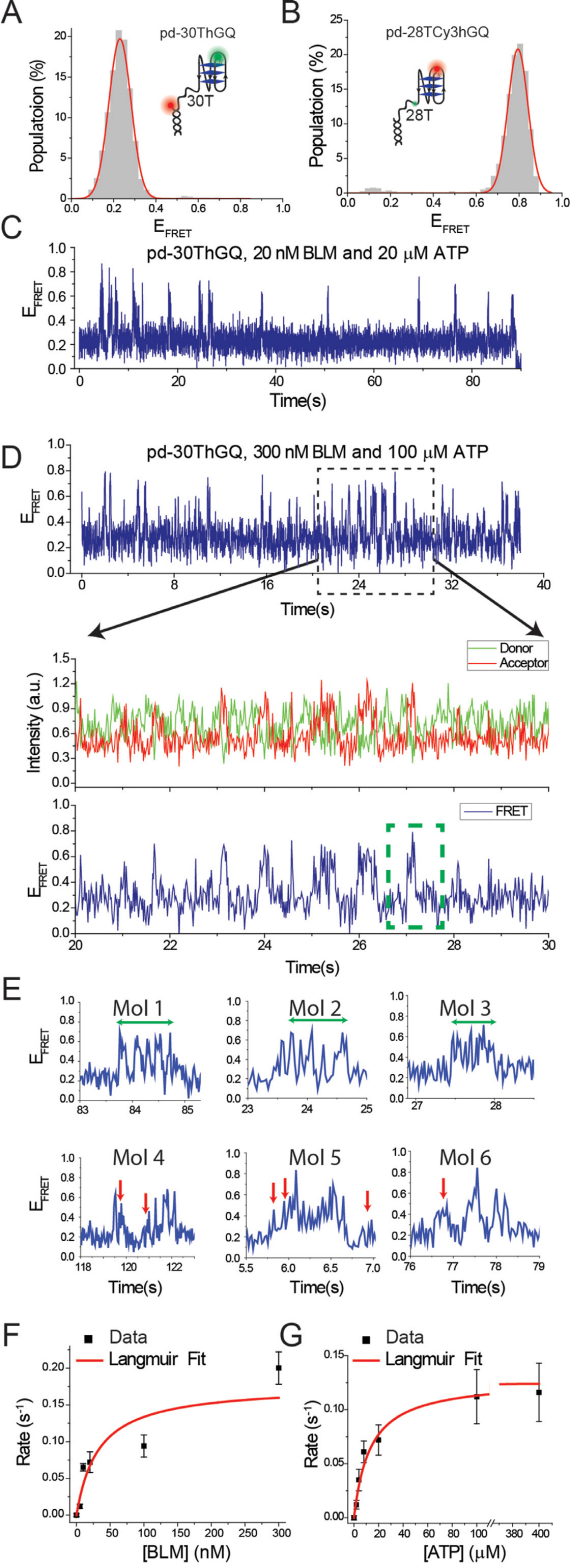
(**A**) smFRET histogram of pd-30ThGQ construct at 150 mM K^+^. The inset shows a schematic of this construct. (**B**) smFRET histogram of pd-28TCy3hGQ construct at 50 mM K^+^. The inset shows a schematic of this construct. smFRET time traces for pd-30ThGQ construct for (**C**) 20 nM BLM and 20 μM ATP; (**D**) 300 nM BLM and 100 μM ATP. (C) and top panel of (D) show FRET signal over an extended time period. These traces show that the reeling events and the subsequent GQ unfolding events become more frequent at higher BLM and ATP concentrations. Middle and bottom panels in (D) show donor-acceptor and FRET signals, respectively, for a narrower time interval in which details of individual events can be identified. In the bottom FRET panel we have indicated a GQ unfolding ‘event’ with a green dashed rectangle. Each event could have several back and forth GQ unfolding-refolding transitions, which we attribute to be due to a single BLM molecule. (**E**) Six sample events are shown to demonstrate the variety of different dynamics that could be observed for BLM–GQ interactions, which we describe in more detail in Results section. Mol 1, Mol 2 and Mol 3 show several back and forth transitions between the high- and low-FRET levels demonstrating several GQ unfolding and refolding events (extended over the green arrows). Mol 4, Mol 5 and Mol 6 show reeling activities that are interrupted due to either releasing of the reeled-in DNA or inability to unfold the GQ, which are marked by red arrows. (**F**) Event rate as a function of BLM concentration at 20 μM ATP. (**G**) Event rate as a function of ATP concentration at 20 nM BLM. The red curves in (F) and (G) are Langmuir binding isotherm fits to the data. The error bars for the event rates in (F) and (G) are determined by dividing the complete data set into three and finding the standard deviation of the mean values of these three data sets.

Having established that BLM can unfold GQ under these conditions, we proceeded with measurements on pd-30ThGQ construct which has a 30T spacer to enable BLM binding and translocation and a GQ-forming sequence in the overhang (see inset of Figure 2A for a schematic). The initial FRET level under conditions that favor GQ formation was *E*_FRET_ = 0.23 ± 0.05 (Figure 2A). In order to confirm stable GQ formation when such a long ssDNA overhang is placed in the vicinity of GQ, we designed the pd-28TCy3hGQ construct which is identical to pd-30ThGQ except that the fluorophores were moved to 2 nt on either side of GQ (see inset of Figure 2B for a schematic). The pd-28TCy3hGQ FRET histogram displayed a peak at *E*_FRET_ = 0.79 ± 0.05 (Figure 2B), which indicates stable GQ formation despite the long overhang. Additional control measurements demonstrating stable GQ formation for this construct are described in Supplementary Figure S2 and are consistent with data on similar constructs (26). smFRET traces for the pd-30ThGQ construct at different BLM and ATP concentrations display FRET bursts from *E*_FRET_ ≈ 0.20 to *E*_FRET_ ≈ 0.70–0.85 at non-periodic intervals (Figure 2C and D). A FRET rise of this nature suggests that BLM bound to the vicinity of ssDNA/dsDNA junction might be pulling the 5′ end toward the junction, where the acceptor fluorophore is located. Such activity was previously observed by PcrA and Pif1 helicases, and it was interpreted to result from these helicases anchoring to the junction and repetitively reeling and releasing the ssDNA overhang. This interpretation is partially consistent with our observations while differing in terms of processivity of the helicase, which will be discussed in more detail. It should be pointed out that certain GQ unfolding events in which BLM binds to the vicinity of GQ, rather than ssDNA/dsDNA junction, may not result in a significant FRET rise, due to lack of reeling activity. Such events may not be registered in our analysis as unfolding of the GQ would result in a lowering the FRET efficiency, which is already low before GQ unfolding and BLM binding (*E*_FRET_ = 0.23 ± 0.05) take place.

After reeling the 30T spacer, BLM should encounter the GQ. At this step the smFRET traces display several dips and rises before the FRET stabilizes at the initial level. Each cluster of these dips and rises is isolated from other such clusters. Each of these clusters will be referred to as an unfolding ‘event’ for analysis purposes. Several examples showing details of these events are shown in Figure 2E. We observed this pattern at all the BLM and ATP concentrations we studied, two examples of which are shown for 20 nM BLM + 20 μM ATP (Figure 2C) and 300 nM BLM + 100 μM ATP (Figure 2D). We interpret the dips in FRET within these clusters as unfolding of the GQ. The rise in FRET that follows a dip could be due to refolding of the GQ or reeling in the newly available ssDNA after the GQ is unfolded.

Having established that BLM can unfold GQ after reeling in the ssDNA between the junction and the GQ, we sought to quantitatively characterize these events as a function of BLM and ATP concentrations. We used the following criteria to identify a FRET burst as an event that includes GQ unfolding. The first criteria is the pattern described earlier, a FRET rise followed by at least one significant dip and rise in FRET. We also required a minimum FRET level (*E*_FRET_ = 0.60) to be reached during the FRET rise to ensure that ssDNA reeling has completed. This threshold was based on separate observations in which FRET rises to *E*_FRET_ < 0.60 followed by a return to the initial FRET level and is consistent with our data on pd-35T, which will be discussed below. Such events were classified as unsuccessful attempts at unfolding GQ, i.e. BLM reels in some or all of the 30T spacer DNA but either dissociates or releases the DNA before unfolding the GQ. Figure 2E shows smFRET traces that demonstrate examples of the rich dynamics of these interactions. The exact descriptions of all the observed features in these traces are not critical for the purpose of this study, but are presented merely to demonstrate the wide spectrum of features in these interactions. Nevertheless, some features that are frequently observed have been identified as signatures of particular processes. For example as mentioned earlier, many traces show dips in the FRET level interfering with the FRET rise. Examples of these events are visible in the traces shown in Figure 2E. Such dips could have different sources including releasing of the reeled-in DNA by BLM before GQ is reached, or unfolding of the GQ after the spacer is reeled in, or possibly dissociation of one BLM molecule from DNA before another one binds and restarts the process that eventually results in unfolding of the GQ.

Event rate analyses (number of unfolding events per second) for different BLM or ATP concentrations, performed based on these criteria, are shown in Figure 2F and G and Supplementary Table S2. The data shown in Figure 2F and G were fit by Langmuir binding isotherm and the resulting fit parameters are listed in Table 1. Each event includes reeling of the poly-T overhang followed by unfolding of the GQ one or more times. We observed that the event rate increased with BLM and ATP concentrations. These clusters of back and forth transitions are isolated from neighboring events and we believe each cluster is due to a single BLM molecule. However, different clusters are considered to be due to different BLM molecules as evidenced by the increase in the event rate with BLM concentration. As each cluster only contains a few successive GQ unfolding events, each BLM molecule unfolds the GQ only a few times before releasing the reeled-in DNA or dissociating from it. These results are different from those observed for Pif1 in terms of protein processivity as Pif1 showed at least an order of magnitude more of such successive GQ unfolding events before it dissociated from DNA (24).

**Table 1.** Summary of Langmuir binding isotherm fit parameters. Brackets [] indicate concentration

			${\rm y} = {\rm A}\;{\rm x}/({\rm x} + {\rm K}_{{\rm eq}} )$		
Construct	Titration co-factor	Fixed parameter	K_eq_	A (Event rate s^−1^)	Corresponding Figure/Table
pd-30ThGQ	[BLM]	20 μM ATP	32.5 ± 21.6 nM	0.18 ± 0.07	Figure 2D, Table S2
pd-35T	[BLM]	20 μM ATP	86.2 ± 16.4 nM	0.35 ± 0.02	Figure 3C, Table S3
pd-12ThGQ	[BLM]	20 μM ATP	140.7 ± 38.7 nM	0.48 ± 0.08	Figure 4D, Table S5
pd-30ThGQ	[ATP]	20 nM BLM	13.3 ± 3.7 μM	0.12 ± 0.02	Figure 2E, Table S2
pd-35T	[ATP]	20 nM BLM	15.2 ± 3.8 μM	0.17 ± 0.01	Figure 3D, Table S3
pd-50T	[ATP]	20 nM BLM	8.7 ± 1.5 μM	0.17 ± 0.01	Figure 3D, Table S4
pd-12ThGQ	[ATP]	100 nM BLM	23.7 ± 7.7 μM	0.29 ± 0.04	Figure 4E, Table S5

### BLM reels in a 5′ tailed polythymine overhang

**Figure 3.**
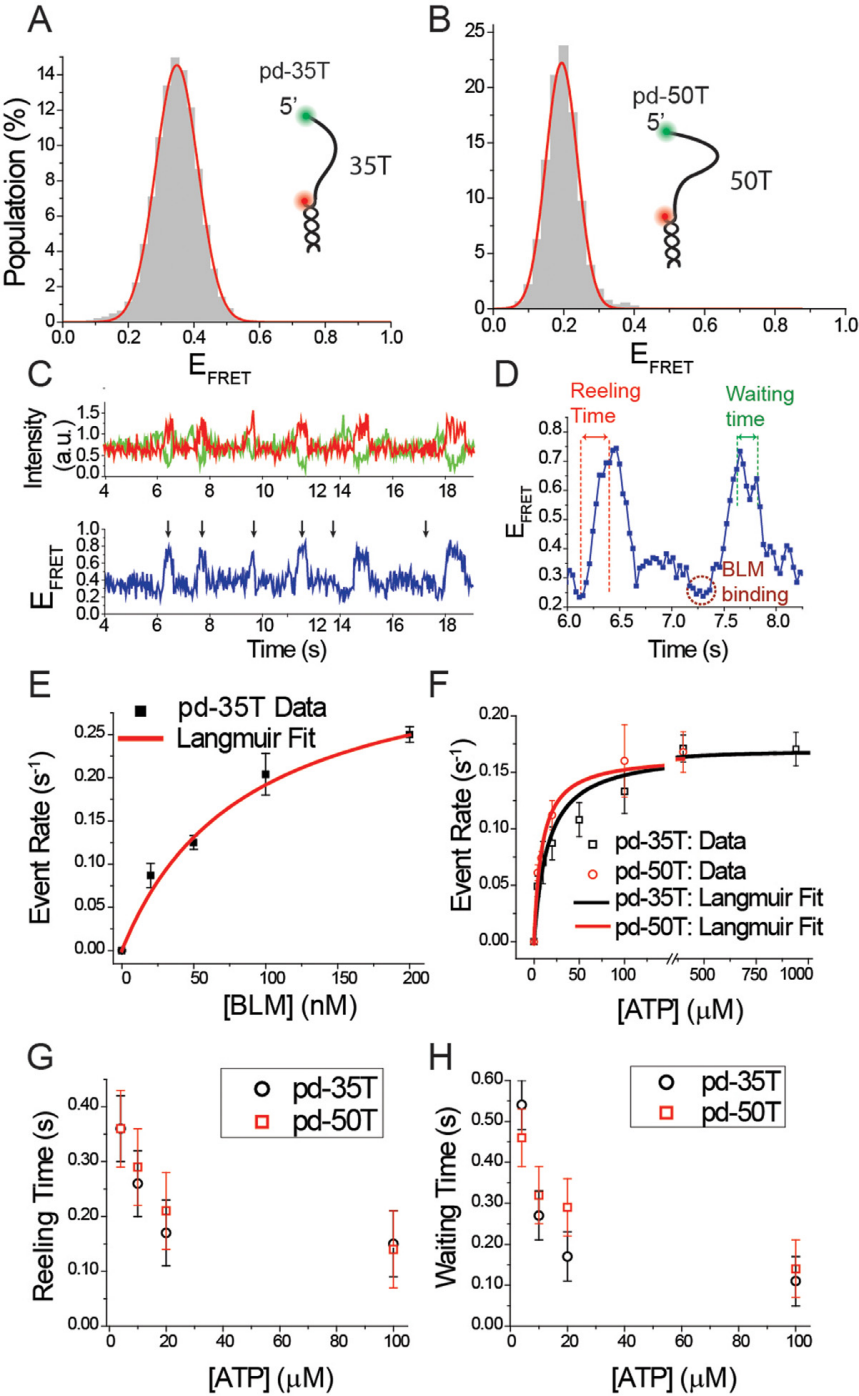
(**A**) smFRET histogram of pd-35T construct at 150 mM K^+^. The inset shows a schematic of this construct. (**B**) smFRET histogram of pd-50T construct at 150 mM K^+^. The inset shows a schematic of this construct. (**C**) A representative smFRET trace showing reeling activity for pd-35T construct. Individual reeling events are marked by black arrows. (**D**) Zoomed-in portion of (C) between times 6.0 s and 8.5 s in which reeling time and waiting time along with BLM binding (initial FRET dip before FRET increase) are identified. (**E**) Reeling event rate as a function of BLM concentration at 20 μM ATP for pd-35T. (**F**) Reeling event rate as a function of ATP concentration at 20 nM BLM for pd-35T (black rectangles) and pd-50T (red circles). The curves in (E) and (F) are Langmuir binding isotherm fits to the data. The error bars for the event rates in (E) and (F) are determined by dividing the complete data set into three and finding the standard deviation of the mean values of these three data sets. (**G**) Reeling time as a function of ATP at 20 nM BLM for pd-35T (black circles) and pd-50T (red rectangles). (**H**) Waiting time as a function of ATP at 20 nM BLM for pd-35T (black circles) and pd-50T (red rectangles). The error bars in (G) and (H) originate from the uncertainty in determining the beginning and end of the reeling time and waiting time, respectively.

Next, we sought to isolate the reeling activity and probe whether it would be observed in the absence of GQ, since such an activity had not been observed for BLM for any type of DNA substrate. Two DNA substrates, pd-35T and pd-50T, which are composed of a duplex DNA stem and a poly-T overhang of 35T or 50T, respectively, were used for these studies. pd-35T and pd-50T have similar overhang length as the combined length of poly-T spacer and GQ-forming sequences of the GQ forming constructs pd-12ThGQ and pd-30ThGQ, respectively. At 150 mM K^+^, pd-35T showed a peak at *E*_FRET_ = 0.35 ± 0.06 (Figure 3A). When BLM and ATP were added, smFRET traces showed non-periodic bursts that rose to *E*_FRET_ ≈ 0.70–0.85 from the starting FRET level of *E*_FRET_ ≈ 0.35 (indicated by arrows in Figure 3C), which are similar to the reeling events observed for pd-30ThGQ. Each of these burst events showed a rapid FRET increase followed by a brief wait at the high-FRET level and then a rapid return to the original FRET level. Figure 3D shows an enlarged version of a smFRET trace between 6.0 and 8.5 s, where the details of two such events are clearly visible. Before the FRET rise starts, a small but noticeable FRET dip (dashed circle) is observed in each event, which is indicative of BLM binding to pd-35T DNA. This small dip is consistent with the FRET level observed for BLM-bound pd-35T substrate in our earlier work (20). Both the irregularity of the time intervals between successive events and the signature of BLM binding to DNA before the reeling-in starts suggest that the same BLM molecule is not necessarily responsible for the different reeling events observed within a single smFRET trace.

The reeling events of these poly-T overhangs did not show the dips and rises after the highest FRET level was reached which were observed for the pd-30ThGQ constructs. This enabled us to characterize not only the event rate, but also the time it takes to rise to the highest FRET (will be referred to as reeling time) and the time it waits at the highest FRET level (will be referred to as waiting time). The reeling time is expected to decrease with increasing ATP concentration. The waiting time is a measure of the time BLM holds on to the reeled-in DNA before either dissociating from it or just releasing it, while still remaining bound to the overhang. There were variations in the shape of the FRET traces among different events. While some had a well-defined high-FRET state, others showed variations in the high-FRET state as shown in the bottom panel of Figure 3C. When determining the waiting time for these different events, we measured the time between completion of the FRET rise and the start of the systematic drop in FRET. Figure 3D shows an example for how the waiting time is measured for an event that shows some variation in the high-FRET state before the FRET sharply drops.

BLM titration measurements were performed at a constant 20 μM ATP concentration on pd-35T construct. The event rates are reported in Figure 3E and Supplementary Table S3. The red curve in Figure 3E is a Langmuir binding isotherm fit to the event rate and the resulting fit parameters are listed in Table 1. Supplementary Figure S3 shows representative smFRET traces showing an increase in the event rate with increasing BLM concentration. These data suggest that BLM does not reel in ssDNA in a processive manner, most likely due to dissociation from DNA. This is in contrast to PcrA and Pif1 which could repetitively reel in the DNA hundreds of times before dissociating from it independent of helicase concentration (24,27). In the case of BLM, we almost exclusively observed a single reeling event before BLM dissociated. To provide a direct comparison to processive helicases such as PcrA and Pif1, we performed a buffer exchange experiment in which the DNA sample was incubated with 1 μM BLM for 15 min, followed by purging the chamber with a buffer that contains ATP but not BLM. Under these conditions, only BLM that is bound to DNA will remain in the chamber and any observed repetitive activity would be attributed to a single BLM molecule. Unlike the processive helicases that showed many repetitive events under these conditions, BLM did not show any reeling activity (data not shown) probably due to its dissociation from DNA. Based on these observations, we conclude BLM performs a reeling activity on these poly-T overhangs non-processively, or possibly with very low processivity if the rare successive reeling events mentioned above are considered.

We further studied the ATP dependence of the event rate (Figure 3F), reeling time (Figure 3G) and waiting time (Figure 3H) at a constant 20 nM BLM concentration for pd-35T construct. Sample smFRET traces at different ATP concentrations are shown in Supplementary Figure S4. The results of these experiments are given in detail in Supplementary Table S3, and Langmuir binding isotherm fit parameters are listed in Table 1. We observed that the event rate increased with increasing ATP concentration. This is considered to be due to BLM being more likely to reel in the DNA once it binds in the vicinity of the junction at higher ATP concentration. More frequent BLM dissociation at higher ATP, which allows binding of another BLM molecule once reeling in is completed, could be another contributing factor for this. As BLM is known to more likely dissociate from DNA in the ADP state (28), a faster rate of ATP hydrolysis would also make dissociation more likely. Both reeling time and waiting time decreased with increasing ATP concentration (Figure 3G and H and Supplementary Table S3). The reeling and waiting times were determined based on a Gaussian fit to a histogram of 300–400 measurements for each case. The errors were estimated based on the uncertainty in determining the beginning and end of the reeling or waiting time, which we estimate to be two data points. Our image acquisition time for these data is 0.032 s. Based on this, the two data point error is 0.064 s, which we round up to 0.07 s. This error estimate is larger than the standard errors obtained from the Gaussian fits, and is a better representative of the resolution of these measurements as it takes into account possible systematic errors that might take place in determining the beginning and end of reeling or waiting time. The decrease in reeling time can be readily understood as an increased rate of BLM reeling in the overhang ssDNA at higher ATP concentrations. The decrease in waiting time suggests BLM either dissociates from DNA or releases the reeled-in DNA more frequently at higher ATP.

Similar results were obtained with the pd-50T construct, which has a 50T overhang (see inset of Figure 3B for a schematic). pd-50T showed an initial *E*_FRET_ = 0.19 ± 0.04 at 150 mM K^+^, as shown in Figure 3B. Supplementary Figure S5 shows sample smFRET traces at different ATP concentrations at 20 nM BLM using pd-50T. A similar pattern of increasing reeling event rate is observed as ATP concentration is increased (Figure 3F and Table 1). In agreement with the pd-35T results, the reeling time and waiting time also decreased with increasing ATP for pd-50T (Figure 3G and H and Supplementary Table S4). Event rates at corresponding ATP concentrations were remarkably similar for the pd-35T and pd-50T constructs, which suggests that BLM has similar binding affinity for both substrates. We note that the reeling time and waiting time parameters for this construct were generally slightly greater than those of pd-35T, although the difference was within the uncertainties associated with measurements and analysis. This could be due to the small difference in the overhang length of these constructs.

### Reeling activity is not required for BLM-mediated GQ unfolding in the presence of ATP

**Figure 4.**
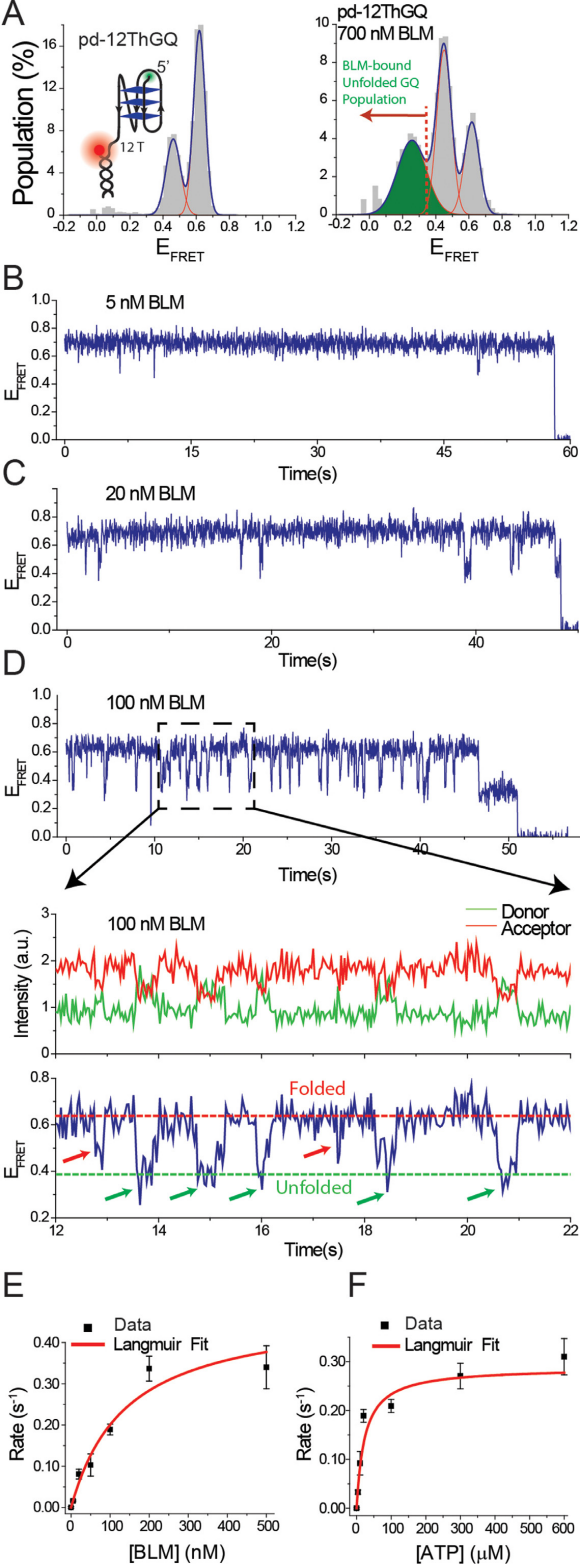
(**A**) (Left Panel) smFRET histogram of pd-12ThGQ construct at 150 mM K^+^. The inset shows a schematic of this construct. (Right Panel) smFRET histogram of pd-12ThGQ when 700 nM BLM is added to the chamber in the absence of ATP. The newly emerging low-FRET peak represents the BLM-bound unfolded GQ state (green peak). The red dashed line and the red arrow indicate the threshold *E*_FRET_ that is used to identify successful GQ unfolding events by BLM. (**B**–**D**) smFRET time traces for pd-12ThGQ construct at 20 μM ATP and (B) 5 nM BLM; (C) 20 nM BLM; (D) 100 nM BLM. The bottom panel in (D) shows a zoomed-in version of a shorter time interval that illustrates the details of the dynamics of the system. The folded and unfolded GQ FRET levels are marked with dashed lines. Five successful GQ unfolding events (marked with green arrows) and two unsuccessful attempts (marked with red arrows) are indicated on the smFRET trace. (**E**) Event rate as a function of BLM concentration at 20 μM ATP. (**F**) Event rate as a function of ATP concentration at 20 nM BLM. The red curves in (E) and (F) are Langmuir binding isotherm fits to the data. The error bars for the event rates in (E) and (F) are determined by dividing the complete data set into three and finding the standard deviation of the mean values of these three data sets.

In order to study BLM-mediated GQ unfolding in the absence of any significant ssDNA reeling, we performed similar measurements on the pd-12ThGQ construct, which is identical to pd-30ThGQ except that it has a shorter spacer of 12 thymines. This shorter spacer almost completely eliminates the reeling activity as it is a few nucleotides longer than the 7 nt footprint of BLM (29). pd-12ThGQ folds into two distinguishable GQ conformations at 150 mM K^+^ as indicated by peaks at *E*_FRET_ = 0.46 ± 0.04 and *E*_FRET_ = 0.62 ± 0.05 ­­­­(Figure 4A, Left Panel). Adding 700 nM BLM to the chamber in the absence of ATP while keeping the ion concentration at 150 mM K^+^ results in the emergence of a new peak at *E*_FRET_ = 0.26 ± 0.09 (green shaded), which represents the BLM-bound unfolded GQ state (Figure 4A, Right Panel). The unfolded GQ state, in the absence of BLM, should yield an FRET peak similar to that of pd-35T (*E*_FRET_ = 0.35, Figure 3A) as the two constructs have identical overhang lengths of 35 nt. The red dashed line and red arrow in the right panel of Figure 4A indicate the threshold *E*_FRET_ value below which we considered a transition as a successful GQ unfolding event. Due to minimal overlap between this region and the higher FRET peaks, over counting GQ unfolding events is unlikely. However, it is more likely that a fraction of successful GQ unfolding events might be ignored in the statistics as indicated by part of the green shaded peak to be above the threshold value. Figure 4B–D show a series of smFRET traces obtained for a BLM titration at 20 μM ATP for pd-12ThGQ. Due to the short spacer, there would be only a few nucleotides between BLM that is bound to the spacer and the GQ. Thus, we did not observe an initial FRET rise due to reeling activity in pd-12ThGQ traces (Figure 4B–D), unlike the constructs that had longer spacers. Instead, frequent dips to *E*_FRET_ ≤ 0.35 were observed representing unfolding of GQ. Occasionally we observed dips that only reached *E*_FRET_ ≈ 0.40–0.50, which we interpreted as BLM-binding events that did not result in unfolding of GQ. Such events were not included in the analysis, and a cut-off value of *E*_FRET_ = 0.35 was used for a dip to be counted as a BLM-mediated GQ unfolding event. Similar to the other constructs we studied, the frequency of these dips increased with increasing BLM and ATP concentration (Figure 4E and F and Supplementary Table S5). We also measured the transition time (Δ*t*_uf_) from the top FRET level to the bottom FRET level, which is a measure of GQ unfolding time, and we did not observe any ATP dependence. Our measurements resulted in transition times in the Δ*t*_uf_ = 0.17–0.19 s range for 4, 10 and 20 μM ATP. These Δt_uf_ were not significantly different from each other given the uncertainty of our measurements (±0.07 s). The uncertainty in these unfolding times was estimated using the two data point uncertainty that was employed for the reeling and waiting times, which is a more conservative estimate than the standard error of the Gaussian fits to histograms of these transition times. We believe the similarity of the unfolding times at different ATP concentrations is reasonable as unfolding of GQ happens in the form of a collapse of the structure, i.e. removing a few nucleotides from the structure leads to unfolding of GQ, and the time for such a collapse to take place should not depend on ATP concentration. We do not observe a step-wise unfolding of GQ, one strand at a time, as proposed for other proteins (24,30).

## DISCUSSION

All measurements in this study were performed with BLM^642–1290^, also called core-BLM, which comprises the RecQ core, RQC and HRDC domains, but lacks the N-terminal domain of the wild-type BLM. This N-terminal domain is involved in multimerization of BLM therefore, core-BLM functions as a monomer. Even though, full length BLM is capable of forming multimers, recent studies have shown that, with rare exceptions, BLM functions as a monomer while unfolding DNA secondary structures (31). In addition, Gyimesi *et al*. showed that contrary to earlier proposed ring-forming oligomerization models of BLM, the majority of BLM proteins remain as monomers under all conditions tested in their study for substrates representing different intermediates of homologous recombination (32). Therefore, monomeric form of BLM has gained broad acceptance as the physiologically relevant stoichiometry.

**Figure 5.**
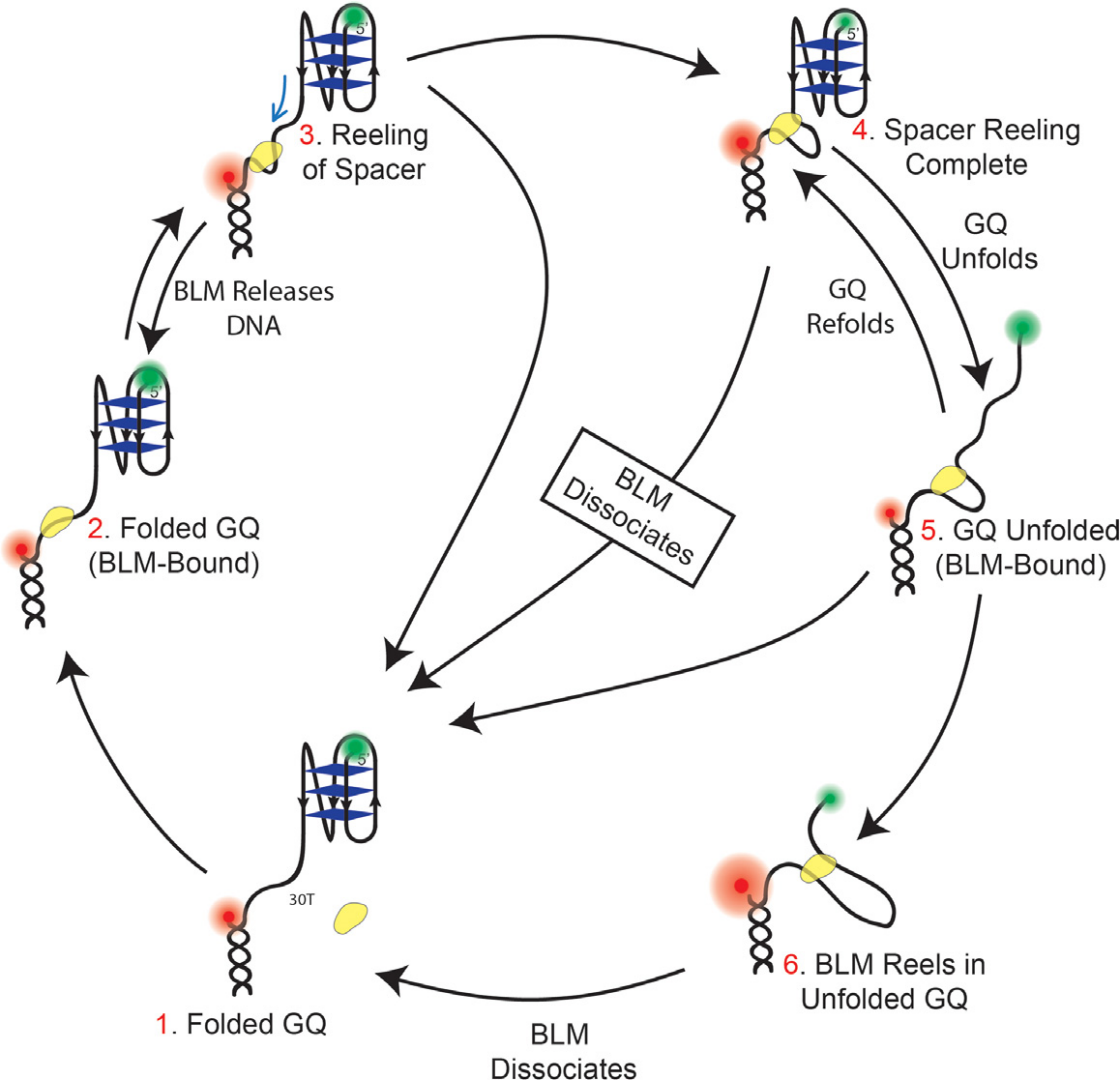
A model describing different phases of BLM-GQ interactions and the underlying dynamics for a substrate with a long DNA spacer (e.g. 30T spacer in pd-30ThGQ) in the presence of ATP. Step 1: The GQ is folded and BLM is not bound to the 30T spacer. Step 2: BLM binds to the spacer while GQ is still folded. Step 3: BLM reels in the spacer which results in an FRET increase. Step 4: Reeling of the spacer is complete and BLM interacts with GQ. At this point several pathways could be followed. BLM could dissociate from DNA without unfolding the GQ or it could release the reeled-in spacer without dissociating from DNA. Both of these would result in a large FRET decrease. Alternatively, BLM could unfold the GQ which would lead to Step 5. Similarly, GQ unfolding should result in a FRET decrease although significantly smaller than what would be observed in the other two alternatives. Step 6: BLM reels in the ssDNA that becomes available after the GQ is unfolded. The evidence for this step is the very high *E*_FRET_ levels (*E*_FRET_ > 0.80), we observe in the smFRET traces. Such high *E*_FRET_ would not be observed if the 23 nt of ssDNA that becomes available after GQ is unfolded is at least partially reeled in.

Combined, our results point to a model for how BLM interacts with GQ structures within an ssDNA overhang as schematically shown in Figure 5, encompassing the various phases of BLM-GQ interactions that are observed in the smFRET traces. Our previous results (20) showed that an initiating event for BLM-mediated GQ unfolding is BLM binding to an ssDNA region in the vicinity of GQ, which enables GQ destabilization even in the absence of ATP. In the current study, for poly-T substrates without GQ, we again observe initial binding of BLM which is then followed by reeling-in of the ssDNA overhang and dissociation of BLM. This reeling activity is only observed in the presence of ATP. When GQ is inserted after the poly-T spacer, reeling-in of the spacer in addition to BLM-mediated GQ unfolding and GQ refolding are observed several times in quick succession, which are followed by BLM dissociation from DNA. The frequency of these unfolding events (event rate) increases with ATP and BLM concentration. BLM-mediated GQ unfolding in this system has relatively low processivity, with significant failed attempts at unfolding GQ followed by dissociation of BLM. Despite this, BLM-mediated GQ unfolding is significantly more efficient with ATP present compared to the case without ATP. As we reported in our previous study, we did not observe significant GQ unfolding in the absence of ATP at 150 mM K^+^ for pd-12ThGQ substrate even at 1 μM BLM concentration (Figure 7 of (20)). The salt concentration had to be reduced to 50 mM K^+^ to observe significant GQ unfolding in 1 μM BLM in the absence of ATP. On the other hand, we observed GQ unfolding for the same DNA construct at 150 mM K^+^ even at 20 nM BLM in the presence of ATP (Figure 4 of this study), demonstrating the ATP-dependent increase in BLM-mediated GQ unfolding efficiency.

A comparison of event rates for substrates that contain GQ versus those that have just a poly-T overhang demonstrates significant reduction in the event rate for the GQ-containing substrates. At 20 nM BLM and saturating ATP concentration both pd-35T and pd-50T display event rates of 0.17 ± 0.01 s^−1^ based on a Langmuir binding isotherm fit. The corresponding rate under similar conditions for the pd-30ThGQ substrate is 0.12 ± 0.02 s^−1^. Given the similar overall spacer lengths for these substrates, we conclude that about 70% of all reeling events result in unfolding of the GQ. A similar comparison with the BLM titration at 20 μM ATP shows saturation rate of 0.35 ± 0.02 s^−1^ for pd-35T while the corresponding rate for pd-30ThGQ is 0.18 ± 0.07 s^−1^, suggesting about 50% of reeling events result in GQ unfolding. This reduction in the event rate would result in an overall slowdown of BLM activity as it would have to restart the reeling activity after failed attempts at unfolding GQ, which are often accompanied by dissociation of BLM from the DNA. A recent study showed a related effect on Pif1 helicase in which non-productive ATP hydrolysis events which failed to result in GQ unfolding were observed (33).

Similar reeling activities displaying significantly more repetitions, were reported for *Bacillus subtilus* PcrA (27) and *Saccharomyces cerevisiae* Pif1 (24). The reeling activity was followed by unfolding a downstream GQ numerous times by the same Pif1 molecule. This activity was suggested to keep the replication fork free of possible secondary structures (34). The highly repetitive nature of Pif1-mediated GQ unfolding correlates with Pif1 being a very efficient destabilizer of GQ. Compared to Pif1, BLM dissociates from DNA much more frequently and hence has much lower processivity. These observations suggest that helicases from different families may utilize similar mechanisms to unfold GQ in the presence of ATP, although with different processivities. A comparison of Pif1 study (24) and our BLM data suggest that dissociation of the helicase from ssDNA overhang is a significant factor in determining its efficiency in unfolding GQ.

Our data in Figure 1 demonstrate that a 2 nt spacer between the GQ and the 12 bp duplex DNA is adequate for BLM to unfold the GQ in the presence of ATP. Similarly, we demonstrated previously (20) that a 2 nt spacer between GQ and the duplex region is adequate for BLM-mediated GQ unfolding in the absence of ATP. These observations are in contrast to a recently published article by Chatterjee *et al*. (21) which reported that GQ unfolding in both the absence and presence of ATP required a minimum of 5 nt spacer between the 5′ end of the GQ and the duplex stem. Based on these and other observations, they proposed a model which suggested that HRDC domain of BLM needs to bind between the duplex and GQ for successful unfolding of GQ. In order to clarify these contrasting observations, we designed a construct which does not have any spacer (i.e. zero nts) between the GQ and duplex. We observed BLM-mediated GQ unfolding for this construct as long as the fluorophore (Cy3) is placed at the end of the 3′ overhang (Supplementary Figure S6). In our system, when the fluorophore was placed internally near the GQ, in a manner similar to that in the Chatterjee *et al*. study, much lower activity was observed with or without ATP. This discrepancy may point to a potential problem of using backbone-labeling at an internal site near GQ, precluding translocation of BLM on the tracking strand. In the case of Pif1 (24), GQ unfolding mediated by ssDNA tail reeling required a much longer 40 nt spacer between the duplex and the 3′ side of the GQ, presumably for Pif1 binding. The internal Cy3 was not backbone-labeled in that study that showed very efficient GQ unfolding by Pif1. These observations indicate that the spacer requirements may be helicase-specific and detection of GQ unfolding may depend on the labeling method and position relative to GQ, the spacer length and ssDNA strand polarity.

We demonstrated that human BLM can bind in the vicinity of ssDNA/dsDNA junction and reel in both an unstructured overhang and an overhang that contains a GQ in the presence of ATP. BLM can unfold GQ after reeling in an ssDNA spacer however, the reeling activity is not required for GQ unfolding, as our data on a construct (pd-12ThGQ) with a short spacer demonstrate. BLM-mediated GQ unfolding and GQ refolding repeat several times before BLM dissociates from DNA. GQ unfolding was observed in 50–70% of the reeling attempts. In the other cases, BLM either released the reeled-in DNA, while remaining bound in the vicinity of junction, or dissociated from DNA completely before it could unfold GQ. Such unsuccessful attempts would naturally slow down BLM activity as each unsuccessful attempt would require restarting the process. These observations are similar in nature to those observed with Pif1 helicase, with BLM being significantly less processive in its repetitive patrolling and GQ unfolding activities. Despite the variations in processivity, the common features observed for the two helicases support the view that different helicases might use similar mechanisms for unfolding GQ structures.

## SUPPLEMENTARY DATA

Supplementary Data are available at NAR Online.

SUPPLEMENTARY DATA
