# Supplementary material for: ATP-dependent G-quadruplex unfolding by Bloom helicase exhibits low processivity
Source: Nucleic Acids Res. 2015 May 18;43(12):5961–70. doi: 10.1093/nar/gkv531 (PMC4499149; doi:10.1093/nar/gkv531)
Supplement: SUPPLEMENTARY DATA [file supp_43_12_5961__index.html]

ATP-dependent G-quadruplex unfolding by Bloom helicase exhibits low processivity — ATP-dependent G-quadruplex unfolding by Bloom helicase exhibits low processivity — SUPPLEMENTARY DATA 

# ATP-dependent G-quadruplex unfolding by Bloom helicase exhibits low processivity

## SUPPLEMENTARY DATA

- SUPPLEMENTARY DATA
