# Supplementary material for: ATP-dependent G-quadruplex unfolding by Bloom helicase exhibits low processivity
Source: Nucleic Acids Res. 2015 May 18;43(12):5961–70. doi: 10.1093/nar/gkv531 (PMC4499149; doi:10.1093/nar/gkv531)
Supplement: SUPPLEMENTARY DATA [file supp_gkv531_nar-00992-f-2015-File007.docx]

**Supplementary Data**

**ATP-dependent G-Quadruplex Unfolding by Bloom Helicase Exhibits Low Processivity.**

Jagat B. Budhathoki^1^, Edward Stafford^1^, Jaya G. Yodh^2,*^, Hamza Balci^1,*^

^1^ Department of Physics, Kent State University, Kent, OH, 44242, USA

^2^ Department of Physics and Center for the Physics of Living Cells, University of Illinois at Urbana-Champaign, Urbana, IL, 61801, USA

*Correspondence should be addressed to hbalci@kent.edu or jyodh@illinois.edu.

**MATERIALS AND METHODS**

**DNA and Protein Constructs**

Partial duplex DNA (pdDNA) constructs with an 18 base-pair (bp) stem and single-stranded DNA (ssDNA) overhang were used for these studies. Figures 1-4 in the manuscript show schematics of the DNA constructs used in this study. The ssDNA overhang either contained a GQ-forming sequence or a non-structured polythymine (poly-T) sequence. The GQ-forming DNA constructs had the human (h) telomeric sequence, (GGGTTA)_3_GGG on the 5’ side, as well as 12 or 30 thymine spacer on the 3' side between the duplex stem and the GQ to provide access for protein binding. The sequences of oligos (purchased from and purified by Integrated DNA Technologies, Coralville, IA) are given in Table S1. The pdDNA constructs were formed by annealing the GQ-containing ssDNA with the DNA-Stem (Table 1), and are called pd-xThGQ with x=12 or 30. In the pdDNA constructs, Cy5 and biotin are on the DNA-Stem and Cy3 is at the flanking 5'-end and is separated from the GQ forming sequence by two thymines. Also utilized was a construct with a duplex at both ends, pd-30ThGQ12bp, which has an 18 bp duplex stem tethered to the surface and a 12 bp duplex at the other end, with an intervening region containing 30 poly-T ssDNA and GQ-forming sequence. When interacting with this construct, BLM has to unfold the GQ sequence in order to be able to unwind the 12 bp sequence. The latter contains the donor fluorophore, so unwinding it releases donor-containing strand resulting in a reduction in the number of donor spots on the surface, and thus providing an indirect probe for BLM-mediated GQ unfolding. In addition to these GQ-forming constructs, two DNA constructs (pd-35T and pd-50T) that do not form any secondary structure were used. These constructs have the same 18 bp duplex stem as the GQ-forming constructs and a ssDNA overhang of either 35 or 50 thymines. These 35 and 50 nt overhangs were selected as they are similar in length to the overhangs of GQ-forming constructs pd-12ThGQ (35 nt total overhang) and pd-30ThGQ (53 nt total overhang), respectively. pd-35T and pd-50T were used for measurements to probe the reeling activity of BLM without the complications that could arise due to GQ formation.

A truncated core BLM (BLM^642-1290^) was purified and characterized as described ([1](#_ENREF_1)). Core BLM lacks the oligomerization domain, and therefore all activity reported in this study is due to monomeric BLM. BLM was stored at a concentration of 2.4 μM in a buffer that contained 50 mM Tris-HCl (pH=7.5), 200 mM NaCl, 50 % (v/v) glycerol and 1 mM DTT. BLM was diluted to the desired concentration in an imaging solution (described in smFRET Assay section) before it was added to the microfluidic channel.

**Sample Preparation**

The sample chamber was created by placing double-sided tape between a quartz slide and a glass cover slip. The drilled quartz slides undergo a rigorous cleaning procedure via acetone and 1M KOH sonication before coating the surface with polyethylene-glycol (PEG) and biotin-PEG in the ratio of ~100:1 (biotin-PEG-5000 and m-PEG-5000 from Laysan Bio Inc.). A pdDNA construct was formed by annealing the ssDNA strand carrying the sequence of interest with an 18 nt long ssDNA oligo, which contained a biotin and the acceptor fluorophore. To illustrate, 12ThGQ and DNA-Stem were mixed to form pd-12ThGQ construct. The two strands were mixed to a final concentration of 1 μM, heated at 90^o^C for 5 minutes, and allowed to cool to room temperature over 2-3 hours. In the particular case of pd-12ThGQ, the annealing conditions significantly influenced the relative population of the two peaks that are observed in smFRET histograms even though the peak positions did not change. When annealed in the absence of any K^+^ in the solution, as was done in all the presented data in this manuscript, the higher FRET peak formed the dominant population. On the other hand, annealing the construct in 10 mM K^+^ resulted in the lower FRET peak dominating the folding histogram. We attribute the two peaks to two different GQ conformations and believe one or the other conformation is preferred based on the annealing conditions. Therefore, it is critical to be consistent with the annealing conditions while working with this construct. Such large variations were not observed in the other constructs we worked with. All experiments were carried out with samples that had undergone annealing the same day. The stock DNA concentration was diluted to 15 pM in a series of three dilutions using 10 mM Tris (pH 7.5) and injected into the chamber, which was previously incubated with neutravidin for 15 min. Excess unbound DNA was washed out by flowing 200 μl of 10 mM Tris (pH 7.5) after 1 minute of DNA incubation. A density of roughly 250 molecules/imaging area (~9×10^3^ μm^2^) is obtained as a result of this protocol.

**smFRET Assay**

smFRET measurements were performed on a prism-based, single-molecule Total Internal Reflection Fluorescence Microscopy (smTIRF) setup, built around an Olympus IX-71 microscope. A green laser (Spectra Physics Excelsior laser with λ=532 nm) was used as the excitation source. Additionally, a red laser (JDS Uniphase He-Ne laser with λ=632.8 nm) was also utilized when direct visualization of acceptor fluorophores was necessary. Depending on the type of measurement and signal strength, data were acquired at 17, 30 or 40 ms/frame integration time using an Andor Ixon EMCCD camera (iXon DV 887-BI EMCCD, Andor Technology,CT).

The imaging solution used in all of the measurements contained Tris base (50mM, pH 7.5), 2 mM Trolox, 0.8 mg/ml glucose, 0.1 mg/ml bovine serum albumin (BSA), 1 mM dithiothreitol (DTT), 0.1 mg/ml glucose oxidase, 0.02 mg/ml catalase, 5 mM MgCl_2_, and 150 mM KCl. As the cation concentration is significant for GQ stability, we henceforth refer to KCl as K^+^ and MgCl_2_ as Mg^2+^. For selected experiments, 50 mM K^+^ or 2 mM Mg^2+^ were alternatively used and indicated as such. BLM and ATP were mixed in the imaging solution at the desired concentration. The imaging buffer was injected into the chamber by a pump operating at 200 μl/min, which enables immediate recording of the data when necessary. In order to ensure proper folding of GQ before introducing BLM, GQ-forming DNA constructs were incubated in the indicated K^+^ concentration (150 mM or 50 mM) for 15 minutes before the protein was added. Proper GQ folding was checked via constructing smFRET histograms before introducing BLM. Long movies (1000-4000 frames) and short movies (30 frames) were recorded for different types of analysis.

| Name | Sequence |
| --- | --- |
| 12ThGQ | 5’- Cy3-TT GGG TTA GGG TTA GGG TTA GGG (12T) **TGG CGA CGG CAG CGA GGC**-3’ |
| 50T | 5’- Cy3-(50T) **TGG CGA CGG CAG CGA GGC**-3’ |
| 35T | 5’- Cy3-(35T) **TGG CGA CGG CAG CGA GGC**-3’ |
| 30T-hGQ-12nt | 5’- **GTA CGA TCG CAG** TT GGG TTA GGG TTA GGG TTA GGG (30T) **TGG CGA CGG CAG CGA GGC**-3’ |
| hGQ4T | 5'-**TGG CGA CGG CAG CGA GGC**TT GGG TTA GGG TTA GGG TTA GGG (4T)-Cy3-3' |
| hGQ11T | 5’-**TGG CGA CGG CAG CGA GGC** GGG TTA GGG TTA GGG TTA GGG (11T)-Cy3-3' |
| 12nt Comp | 5’-Cy3-**CTG CGA TCG TAC** |
| DNA-Stem | 5'-Biotin-**GCC TCG CTG CCG TCG CCA** Cy5-3' |

**Table S1**. The DNA constructs used in the study. The underlined nucleotides form the GQ structure. The number of consecutive thymines are written in parenthesis, e.g. (30T) means 30 consecutive thymines. The bold sections in the top four rows form duplex DNA when annealed with constructs in the bottom two rows (DNA-Stem or 12nt Comp).

**Identification of FRET level of folded and unfolded states**

In our constructs, coiled DNA is stabilized into a GQ structure by K^+^ ions, resulting in a higher FRET in the folded state compared to the unfolded state of GQ forming constructs. Nevertheless, the difference in FRET between the folded and unfolded constructs is fairly small for some of the constructs used in this study due to the relatively long overhangs (~30 nt), composed of poly-T sequences that do not take part in the GQ structure and contribute to the FRET efficiency of both folded and unfolded constructs. As shown in Figure 4, pd-12ThGQ showed two well-separated folded peaks at E_FRET_=0.46±0.04 and E_FRET_=0.62±0.05, which represent two GQ conformations, in agreement with earlier observations for telomeric sequence in 150 mM K^+^ ([2](#_ENREF_2)). On the other hand, pd-30ThGQ construct manifested a folded peak at E_FRET_ =0.23±0.05 (Figure 2). This low FRET peak can be attributed to relatively large separation between Cy3 and Cy5 due to the 30T spacer between 18 bp duplex stem and the G-quadruplex. Nevertheless, the low FRET peak proved to be very practical in this context as reeling of the DNA by BLM results in significantly higher FRET efficiencies (up to E_FRET_=0.70-0.85), which enable clear identification of these events as spikes over a relatively flat background in smFRET traces.

In order to have a reference point for the unfolded state E_FRET_ for these constructs, similar histograms were constructed for pd-35T and pd-50T which do not form GQ (Figure 3). pd-35T showed a FRET peak at E_FRET_=0.35±0.06. This is significantly smaller than both peaks observed for pd-12ThGQ construct, which also has a 35 nt long overhang when poly-T and GQ forming sequences are considered together (21 nt GQ forming sequence and 12+2 nt of thymines). This suggests that pd-12ThGQ construct forms a GQ under these assay conditions. On the other hand, pd-50T shows a peak at E_FRET_=0.19±0.04, which should be compared with the similarly sized pd-30ThGQ (which has a total overhang length of 53 nt (21 nt forming GQ and 30+2 nt of thymines)). Therefore, the FRET efficiency of this construct is expected to be slightly lower than that of pd-50T (which has a 50 nt overhang) when GQ is not folded. Since pd-30ThGQ shows a slightly higher E_FRET_ peak compared to pd-T50 peak (E_FRET_=0.23 *vs.* E_FRET_=0.19), we consider this as evidence for formation of a secondary structure in pd-30ThGQ. The fact that the difference between the two FRET peaks is small is not unexpected due to two reasons. First of all, E_FRET_≈0.20 is a minimally sensitive range for FRET. Hence, significant differences in distance result in relatively small FRET changes. Secondly, pd-30ThGQ contains an unstructured 32 nt long segment (30 nt on 3'-end of GQ and 2 nt on the 5'-end), which contributes to the overall FRET efficiency regardless of whether the GQ is folded or not.

As folding of the GQ under the smFRET assay conditions is of critical importance for all the analysis performed in this study, we performed an additional control to test whether the GQ folds when it has such a long overhang. We maintained an essentially identical design, but pushed the donor-acceptor fluorophores to the ends of the GQ (a 2 nt thymine spacer was maintained on either side to reduce possible interactions between the GQ and the fluorophores). In this construct (pd-28TCy3hGQ), FRET directly probes GQ folding and is not influenced by the long overhang. As expected, a single high FRET population is observed at E_FRET_=0.79±0.05 even at 50 mM K^+^ (Figure 2), suggesting a folded GQ conformation.

**BLM Cannot Unwind the Duplex Without Unfolding the GQ**

In Figure 1 of the manuscript, we used unfolding of the duplex DNA and removal of the donor fluorophore from the surface as evidence for unfolding of the GQ. In order to further strengthen this argument, we directly probed whether BLM can bind to the GQ or make contact with the short spacer (2 nt) between the duplex and GQ and proceed to unwind the duplex without unfolding the GQ. We designed a construct in which GQ has a short enough overhang (4 nt) that BLM cannot bind to it. pd-hGQ4T construct was formed by annealing hGQ4T and the DNA-Stem (Table S1). This construct has a free 3'-end and the duplex is on the 5' side of GQ (Figure S1A). Therefore, the only way for BLM to unwind the duplex is via either binding to the GQ or make contact with the spacer between the GQ and duplex. As the overhang is shorter than the footprint of BLM, we do not expect BLM to be able to unfold the GQ by binding to the overhang. Under these circumstances, we did not observe any unwinding of the duplex DNA at different BLM and ATP concentrations. The fraction of Cy3 spots that remained on the surface essentially decreased at a rate similar to that expected from photobleaching (Figure S1C). Therefore, we conclude that BLM cannot proceed to unwind the duplex DNA by binding to the GQ or the short spacer between the GQ and duplex.


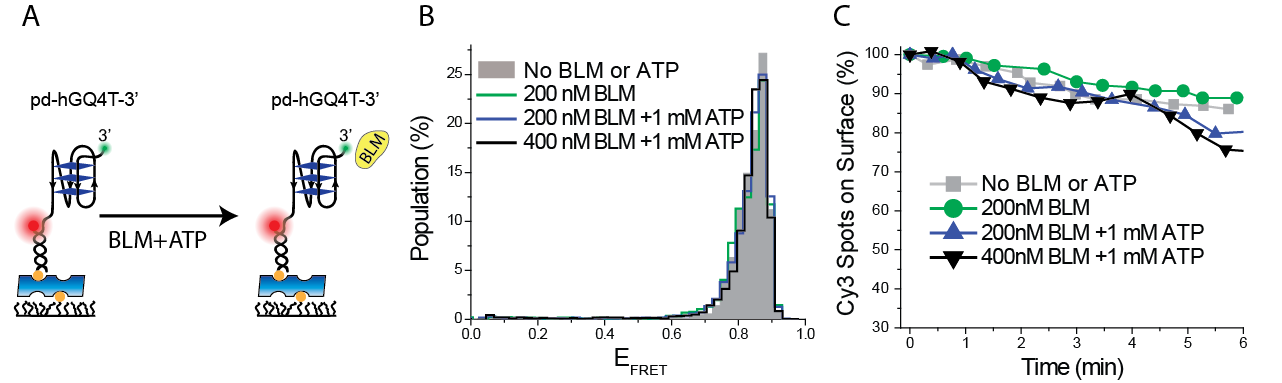
Figure S1. (A) A schematic of the measurement which did not show unwinding of the duplex when the upstream could not be unfolded. (B) SmFRET histograms at different BLM and ATP concentrations. These histograms show that the GQ is not unfolded under any of the BLM and ATP concentrations we studied as the folded GQ peak was the only peak observed in these measurements. (C) The fraction of Cy3 spots that remained bound to the surface at different BLM and ATP concentrations. Essentially all data sets resulted in a similar rate of decrease in the number of Cy3 spots which is consistent with the photobleaching rate that is obtained from the data in the absence of ATP.

**Control Measurements to Probe GQ Formation for pd-30ThGQ**

In smFRET studies, GQ formation is typically confirmed by a prominent rise in the FRET peak upon folding of GQ, which is a significantly more compact structure compared to the random coiled ssDNA. However, due to the long (30 T) spacer between the donor-acceptor fluorophores, which is in random coil conformation regardless of whether GQ is folded or not, such a prominent change in FRET peak is not observed upon folding of GQ in the pd-30ThGQ construct. Therefore, we sought to perform control measurements that would probe GQ formation in a construct that has such a long overhang under our smFRET assay conditions. Previous circular dichroism and thermal melting studies have shown that human telomeric sequence with comparable overhang length folds into GQ even at 50 mM K^+^ ([3](#_ENREF_3)). In addition, studies on Pif1-GQ interactions have utilized constructs with even longer spacers (42 nt) under lower salt concentration (60 mM K^+^) ([4](#_ENREF_4)). As our studies on pd-30ThGQ are performed in 150 mM K^+^ and have a shorter spacer, the formed GQ structures should be even more stable compared to those in references ([3](#_ENREF_3)) and ([4](#_ENREF_4)).

In order to probe GQ formation and stability, we performed complementary strand titration measurements on pd-28TCy3hGQ construct under identical assay conditions as those used for BLM studies. pd-28TCy3hGQ similarly has a 30 nt long spacer but the donor-acceptor fluorophores are each placed 2nt away from the GQ, resulting in a high FRET peak upon folding of GQ (E_FRET_=0.79). In this measurement, CCCTAACCCTAACCCTAACCC strand, which is complementary to GQ forming segment, is titrated in 150 mM K^+^ or 150 mM Li^+^. Duplex formation, which results in a significantly lower peak (E_FRET_=0.12) compared to GQ formation, is taken as signature of elimination of the GQ structure. The fraction of duplex structures that are formed in the presence of a given complementary strand concentration is inversely proportional to GQ stability. As human telomeric GQ is known to be more stable in K^+^ compared to Li^+^, we expect to observe a difference in the fraction of duplex formation in the presence of the two ions. If a GQ is not formed, such a difference is not expected. Figure S2 shows results of this titration measurement. As expected, in the case of 150 mM K^+^, 69±3% of the GQ are converted into a duplex at saturating complementary concentration while 100% of them are converted to duplex in 150 mM Li^+^. These results strongly support stable GQ formation in 150 mM K^+^ for the telomeric GQ with 30 nt long spacer.


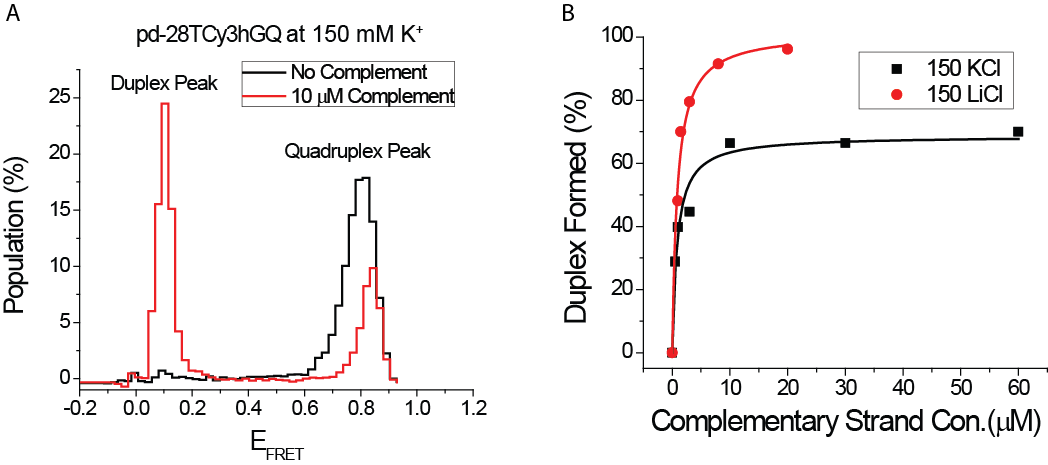


**Figure S2:** Complementary strand titration measurements on pd-28TCy3hGQ construct. (A) Duplex formation results in a significantly different FRET peak compared to the folded GQ. (B) Percentage of duplex population (lower FRET peak in (A)) as a function of complementary strand concentration in 150 mM K^+^ or 150 mM Li^+^. At saturating complementary strand concentration less duplex formation is observed in K^+^ compared to Li^+^, which is indicative of GQ formation.

**Event Rate for pd-30ThGQ as a Function of BLM and ATP Concentration**

| [BLM] (nM) | [ATP] (μM) | GQ Unfolding Events | |
| --- | --- | --- | --- |
|  |  | Rate (s^-1^) | Counts |
| 20 | 2 | 0.012±0.004 | 103 |
| 20 | 4 | 0.035±0.010 | 204 |
| 20 | 8 | 0.061±0.010 | 415 |
| 20 | 20 | 0.072±0.014 | 465 |
| 20 | 100 | 0.112±0.025 | 825 |
| 20 | 400 | 0.116±0.027 | 573 |
| 5 | 20 | 0.012±0.003 | 80 |
| 10 | 20 | 0.065±0.005 | 749 |
| 20 | 20 | 0.072±0.014 | 465 |
| 100 | 20 | 0.094±0.015 | 1096 |
| 300 | 20 | 0.200±0.022 | 511 |

**Table S2:** Number of observed GQ unfolding events and unfolding rate for pd-30ThGQ construct as a function of ATP (at 20 nM BLM-unshaded rows) or BLM (at 20 μM ATP-shaded rows) concentration. Each event includes reeling of the poly-T overhang followed by unfolding of the GQ one or more times. The "Counts" column designate the number of events used for calculating the rate of unfolding events. The errors in the rate are obtained by dividing the entire data set into three sets and finding the standard deviation of the rates obtained from each of these three sets.

**Reeling-in activity is carried out by a single BLM with low processivity**

The smFRET time traces show that the ssDNA overhang can be reeled-in by BLM multiple times in a single trace. In order to investigate whether these reeling events were carried out by a single monomer of BLM repetitively or different BLM monomers, we measured the frequency of these events as a function of BLM concentration. We expected the frequency of reeling events to remain unchanged at different BLM concentrations if the activity in each trace is due to a single BLM monomer. Conversely, we expected the frequency to increase with concentration if the activity is due to multiple BLM monomers, e.g. a monomer dissociates from DNA after completing one or a few reeling events and another monomer binds to DNA and repeats the activity. The pd-35T construct was used for these measurements (see Figure 3 for a schematic of the construct) and BLM was titrated with 20 μM ATP. At 20 nM BLM, well-separated reeling events, identified as rising spikes in the smFRET traces, were observed as shown in Figure S3-A. These events were separated by irregular time intervals. Increasing the BLM concentration to 50 nM and then to 100 nM (Figure S3-B and C) resulted in more frequent events in the observation time of about 2 min. A quantitative summary of event rates at different BLM concentrations is presented in Table S3 and shows that the reeling events do not have high processivity. Whether this activity takes place non-processively or with low processivity is less clear. We decided the low-processivity is a better description partially due the broader range of validity as opposed to non-processive which has a very strict definition that we do not have conclusive evidence for. Our decision was influenced by our observations with the constructs that contain GQ in which we clearly observe that BLM can remain bound in the vicinity of GQ through multiple GQ unfolding/refolding cycles and does not immediately dissociate from the DNA after unfolding the GQ. For these constructs we have observed smFRET traces where BLM reels in the spacer and then reels in ssDNA that becomes available after the GQ is unfolded. This is an example of BLM performing multiple reeling events without dissociating from the DNA. Even though the processes are different in nature for the poly-T and GQ forming constructs, we consider this as an implication of low-processivity.


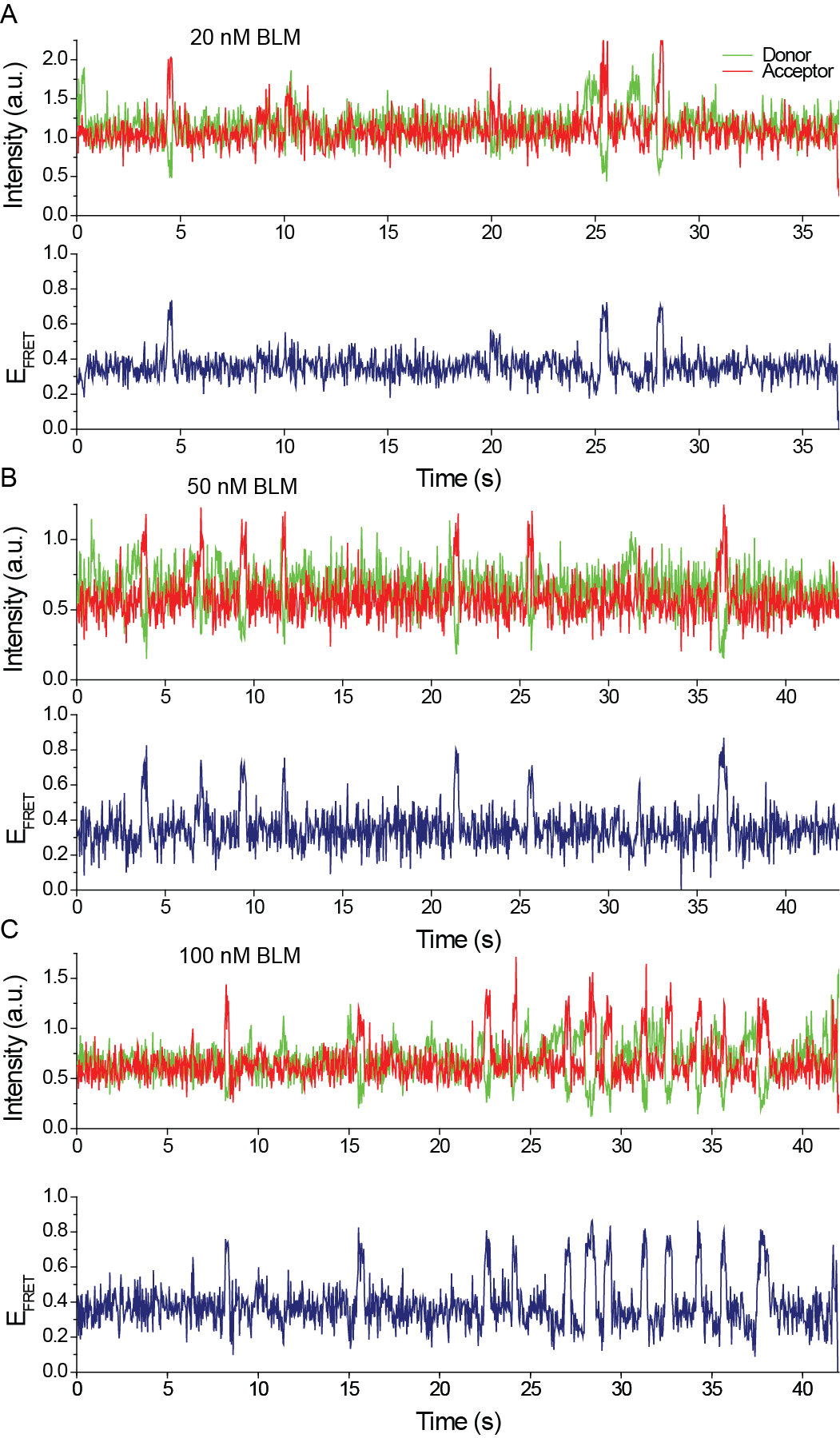


**Figure S3.** smFRET time traces reveal reeling-in activity as a function of BLM concentration at 20 μM ATP for the pd-35T construct. E_FRET_≈0.35 represents initial FRET where DNA is not bound by BLM. FRET gradually increases up to ≈0.85 and drops down to the initial FRET after a brief wait at the high FRET state. These events repeat at a frequency that is dependent on BLM concentration. Sample smFRET traces are shown for (A) 20 nM BLM; (B) 50 nM BLM; (C) 100 nM BLM. Donor and acceptor intensities are shown in the top panel and the corresponding FRET is shown in the bottom panel in A-C.

**ATP Dependence of Reeling Activity for pd-35T Construct**

In order to examine the ATP dependence of the reeling activity, ATP was titrated at a constant BLM concentration (20 nM) using the pd-35T construct. Sample smFRET traces at 4, 20 and 100 μM ATP are shown in Figure S4. As these traces demonstrate, the frequency of events increases with increasing ATP concentration. We believe this ATP dependence is due to two effects: (i) At higher ATP concentrations, a BLM that binds to DNA is more likely initiate a reeling activity; (ii) At higher ATP concentrations BLM is more likely to dissociate from DNA and enable another monomer to bind and repeat the activity. BLM is most likely to dissociate from DNA in the ADP state of ATP hydrolysis cycle ([5](#_ENREF_5)), which is more frequently visited at higher ATP concentration. In addition, both the time it takes to reach the high FRET state and the time BLM spend at this state were determined to be shorter for higher ATP concentrations (Table S3 and Figure 3E-F), which further support this activity to be ATP-dependent.


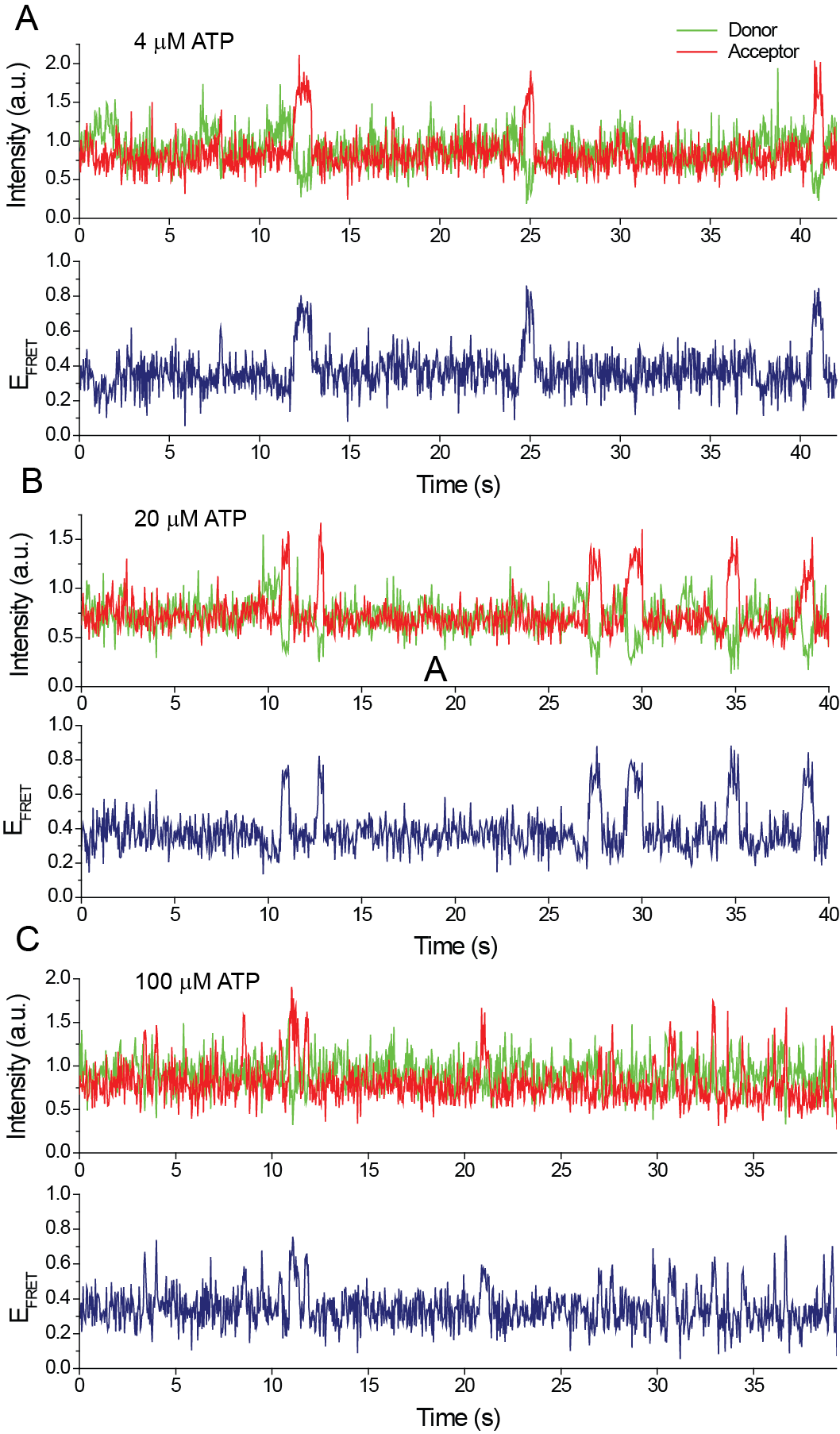


**Figure S4.** smFRET time traces displaying reeling activity as a function of ATP concentration at 20 nM BLM for pd-35T construct. E_FRET_≈0.35 represents initial FRET where BLM is not bound to the DNA. FRET gradually increases up to ≈0.85 and then drops down to its initial level after a brief wait at the high FRET state. These events repeat at a frequency that depends on ATP concentration. Sample smFRET traces are shown for (A) 4 μM ATP; (B) 20 μM ATP; (C) 100 μM ATP. Donor and acceptor intensities are shown in the top panel and the corresponding FRET is shown in the bottom panel in A-C.

**Summary of Data on pd-35T Construct**

| Construct | [BLM]  (nM) | [ATP] (μM) | Reeling Events | | Reeling Time | | Waiting Time | |
| --- | --- | --- | --- | --- | --- | --- | --- | --- |
|  |  |  | Rate(s^-1^) | Counts | Time (s) | Counts | Time (s) | Counts |
| pd-35T | 20 | 4 | 0.049±0.009 | 417 | 0.36±0.07 | 231 | 0.54±0.07 | 317 |
|  | 20 | 10 | 0.070±0.018 | 509 | 0.26±0.07 | 240 | 0.27±0.07 | 268 |
|  | 20 | 20 | 0.087±0.015 | 380 | 0.17±0.07 | 276 | 0.17±0.07 | 256 |
|  | 20 | 50 | 0.108±0.015 | 393 |  | | | |
|  | 20 | 100 | 0.133±0.02 | 413 | 0.15±0.07 | 250 | 0.11±0.07 | 254 |
|  | 20 | 400 | 0.171±0.013 | 776 |  | | | |
|  | 20 | 1000 | 0.178±0.027 | 865 |  |  |  |  |
|  | 20 | 20 | 0.087±0.015 | 380 |  |  |  |  |
|  | 50 | 20 | 0.125±0.004 | 655 |  |  |  |  |
|  | 100 | 20 | 0.204±0.024 | 931 |  |  |  |  |
|  | 200 | 20 | 0.250 ±0.009 | 560 |  |  |  |  |

**Table S3**: Characteristic reeling activity parameters for pd-35T as a function of BLM (at 20 μM ATP-unshaded rows) and ATP (at 20 nM BLM-shaded rows) concentration. The "Counts" columns designate the number of events used for calculating the relevant parameter. The errors in the rate are obtained by dividing the entire data set into three sets and finding the standard deviation of the rates obtained from each of these three sets. The errors in the reeling time and the waiting time are based on the uncertainty in determining the beginning and end of the reeling or waiting, respectively, which we estimate to be 0.07 s.

**ATP Dependence of Reeling Activity for pd-50T Construct**

In order to check the validity of the results as observed for pd-35T, similar measurements were performed on the pd-50T construct. Figure S5 shows example smFRET traces at different ATP concentrations and 20 nM BLM using this construct and Table S4 summarizes the quantitative measurements on the event rate, reeling time, and waiting time. Initial E_FRET_ (before BLM binding) was found to be 0.19±0.04 at 150 mM K^+^ and 5 mM Mg^+2^. A similar pattern of increasing frequency of reeling activity is observed as ATP concentration is increased. In addition to frequency, parameters such as rising time and waiting time at high FRET were also measured and showed a similar ATP dependence as was the case with pd-35T (Table S4). We note that we did not observe a significant difference in rising time for this construct compared to pd-35T, which we attribute to our limitations in terms of time resolution and the associated uncertainties in determining the beginning and end of the rising time which are inadequate to distinguish between these two constructs that have a length difference of only 15 nt. Another possible reason for the similarity is that BLM might be reeling in a certain number of nucleotides before dissociating from DNA or releasing the overhang.


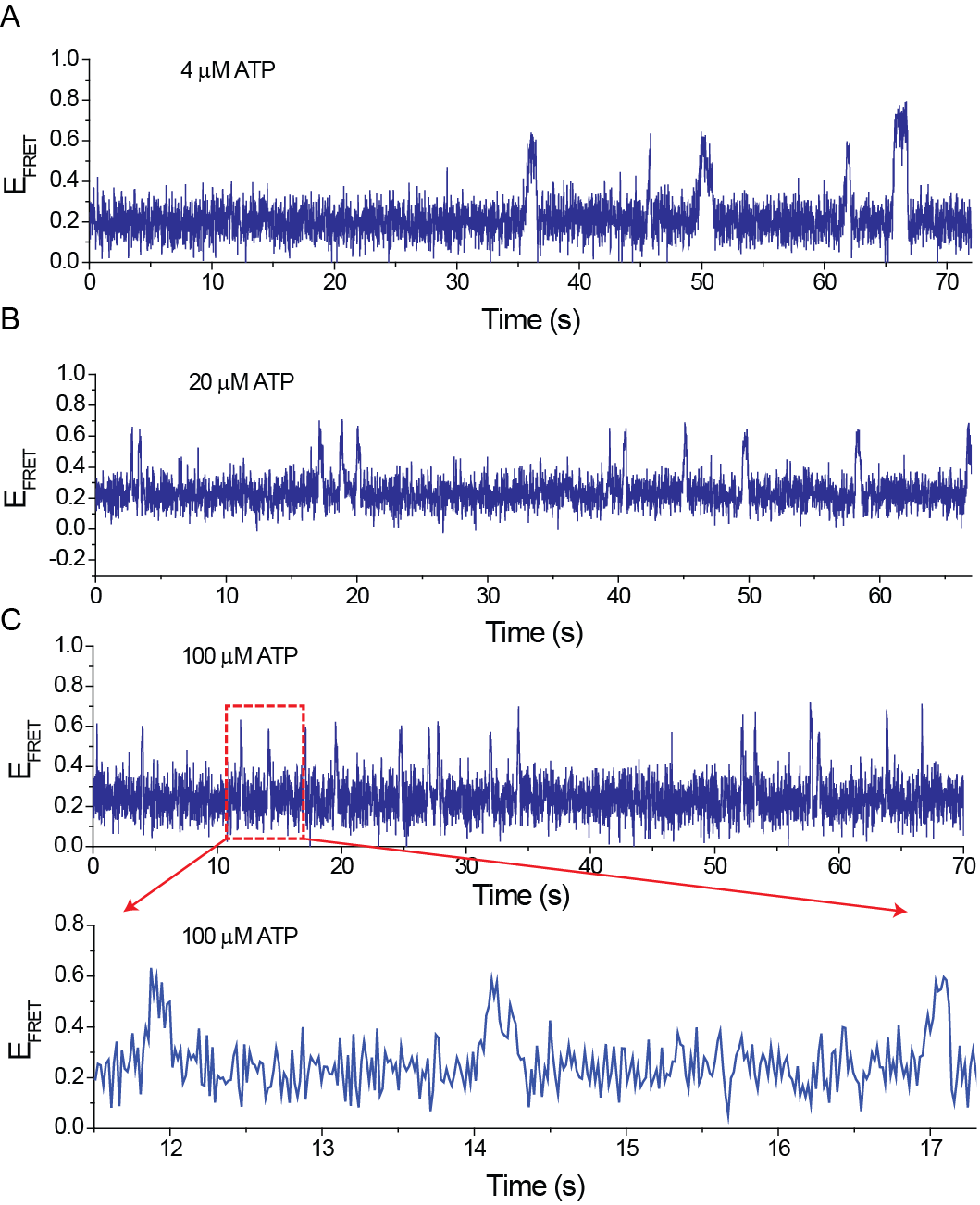


**Figure S5.** smFRET time traces displaying reeling activity as a function of ATP concentration at 20 nM BLM for pd-50T construct. E_FRET_≈0.19 represents initial FRET where BLM is not bound to the DNA. FRET gradually increases up to ≈0.80 and drops down to its initial level after a brief wait at the high FRET state. The event rate increases with ATP concentration as demonstrated by the sample traces. Example smFRET traces are shown for (A) 4 μM ATP; (B) 20 μM ATP; (C) 100 μM ATP. The bottom panel in (C) focuses on a small time interval of the upper panel and shows details of several events.

| Construct | [BLM]  (nM) | [ATP] (μM) | Reeling Events | | Reeling Time | | Waiting Time | |
| --- | --- | --- | --- | --- | --- | --- | --- | --- |
|  |  |  | Rate(s^-1^) | Counts | Time (s) | Counts | Time (s) | Counts |
| pd-50T | 20 | 4 | 0.061±0.007 | 770 | 0.36±0.07 | 252 | 0.46±0.07 | 338 |
|  | 20 | 8 | 0.074±0.006 | 357 | 0.29±0.07 | 255 | 0.32±0.07 | 250 |
|  | 20 | 20 | 0.112±0.013 | 574 | 0.21±0.07 | 352 | 0.29±0.07 | 335 |
|  | 20 | 100 | 0.16± 0.032 | 1487 | 0.14±0.07 | 304 | 0.14±0.07 | 325 |
|  | 20 | 400 | 0.183±0.018 | 581 |  | | | |

**Table S4**: Characteristic reeling activity parameters for pd-50T as a function of ATP concentration. The "Counts" columns designate the number of events used for calculating the relevant parameter. The errors in the rate are obtained by dividing the entire data set into three sets and finding the standard deviation of the rates obtained from each of these three sets. The errors in the reeling time and the waiting time are based on the uncertainty in determining the beginning and end of the reeling or waiting, respectively, which we estimate to be 0.07 s.

**Summary of Data on pd-12ThGQ Construct**

| **[BLM] (nM)** | **[ATP] (μM)** | **GQ Unfolding Events** | |
| --- | --- | --- | --- |
|  |  | **Rate (s^-1^)** | **Counts** |
| 100 | 4 | 0.033±0.002 | 321 |
| 100 | 10 | 0.114±0.015 | 424 |
| 100 | 20 | 0.189±0.013 | 2057 |
| 100 | 100 | 0.209±0.013 | 1243 |
| 100 | 300 | 0.270±0.025 | 723 |
| 100 | 600 | 0.313±0.037 | 621 |
| 5 | 20 | 0.016±0.002 | 141 |
| 20 | 20 | 0.081±0.012 | 473 |
| 50 | 20 | 0.103±0.027 | 575 |
| 100 | 20 | 0.189±0.013 | 2057 |
| 200 | 20 | 0.318±0.068 | 852 |
| 500 | 20 | 0.336±0.052 | 1305 |

**Table S5**: Number of observed GQ unfolding events and the GQ unfolding rate for pd-12ThGQ construct. The shaded rows are BLM titrations at 20 μM ATP, while the unshaded rows are ATP titrations at 100 nM BLM. The "Counts" column designate the number of events used for calculating the rate of GQ unfolding.

**A spacer is not required for BLM to unfold GQ:**

In their recent work, Chatterjee *et al.* proposed a model that suggests the requirement of a 5 nt or longer spacer between partial duplex stem and GQ in order for BLM to be able to unfold the human telomeric GQ at 50 mM K^+^ ([6](#_ENREF_6)). In our earlier work, we worked with human telomeric GQ that have an 6-15 nt long 3' overhang, similar to the construct shown in Figure S6C, and just a 2 nt spacer between the duplex and GQ. We demonstrated that BLM is capable of unfolding these human telomeric GQ constructs that have a 2 nt spacer between the GQ and the duplex at 150 mM K^+^ and in the absence of ATP. One of the differences between our construct and that of Chatterjee *et al.* was the location of the donor (Cy3) fluorophore. We had placed the Cy3 fluorophore at the 3'-end of the GQ forming strand while Chatterjee *et al.* had placed it internally in the vicinity of GQ. In order to further study the discrepancy between the two results, and a possible involvement of the fluorophore location in the discrepancy, we repeated our measurements on a DNA construct that does not have any spacer between the GQ and the duplex, but has the Cy3 at the 3'-end rather than at an internal site (see Figure S6C for a schematic). This construct should directly probe whether a spacer between the GQ and the duplex is required in order for BLM to be able to unfold the GQ. For a direct comparison, we performed this test under identical ionic conditions, and used the same BLM and ATP concentrations as those used by Chatterjee *et al.* The sequence we used was hGQ11T : 5’-**TGG CGA CGG CAG CGA GGC** GGG TTA GGG TTA GGG TTA GGG (11T)-Cy3, where the underlined segment forms GQ and the bold section forms the duplex upon hybridization with DNA-Stem: 5'-Cy5-**GCC TCG CTG CCG TCG CCA**-3'-Biotin. Figure S6-A (red histogram) shows GQ formation by this construct in 50 mM K^+^ and 2 mM Mg^2+^. We first tested whether 20 nM BLM unfolds this GQ in the absence of ATP, and observed significant unfolding under these conditions (Figure S6-A, green histogram). We also performed similar measurements with 20 nM BLM and 4 μM ATP at 50 mM K^+^. We used dissociation of the Cy3-labeled strand from the surface as a probe for GQ unfolding as BLM must unfold GQ before it can unwind the duplex and release that strand (schematic in Figure S6-C). We found that Cy3 spots disappeared in a time-dependent manner as would be expected. The fraction of remaining Cy3 spots is shown in Figure S6-B. Figure S6-C shows images of this experiment at different times. The images on the left and the middle show the system with green excitation at time t=0 s and t=132 s, respectively. The rightmost image was obtained by exciting the same spot with a red laser. The same area of the sample was illuminated with a laser light for 0.5 s followed by turning off the illumination for ≈30 s. This cycle was repeated 14 times (total illumination time of ≈7 s and total time of ≈527 s). As the red excitation (right-most image) demonstrates, most Cy5 spots still remain on surface while a comparison of the left and middle images shows that most of the Cy3 spots have left the surface. Under identical imaging, laser power, and time lapse conditions, less than 10% of the spots have been observed to be eliminated due to photobleaching. We conclude that BLM unfolds the GQ and does not require a spacer between the duplex and quadruplex for this unfolding. Furthermore, we show that BLM can unwind an 18 bp duplex in the immediate vicinity of the GQ at ATP concentrations as low as 4 μM. We attribute the difference between our results and those of Chatterjee *et al.* to the internal label used in their study, which breaks the backbone of the DNA and we believe might interfere with BLM activity.


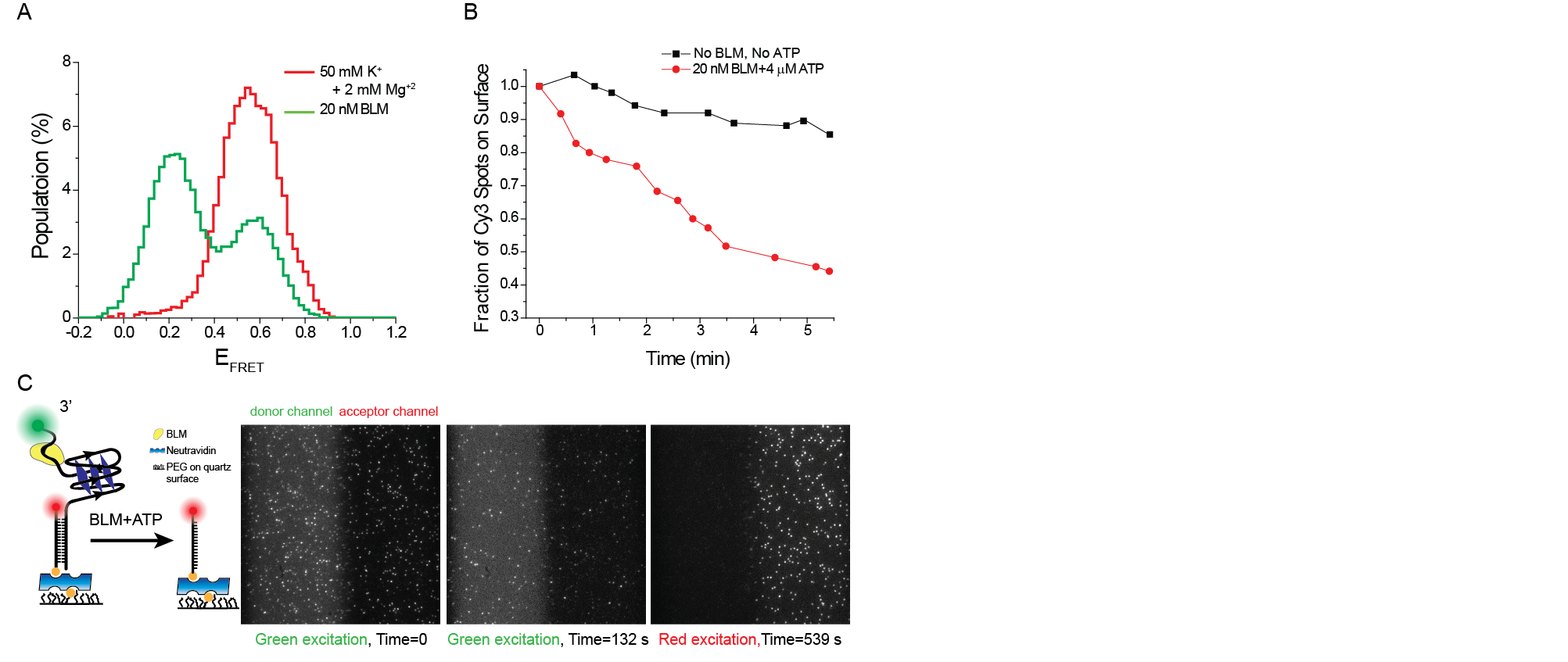


**Figure S6.** smFRET studies on a DNA construct that does not have a spacer between the duplex and quadruplex. A) GQ folds (E_FRET_ ≈0.58) as shown by red histogram in 50 mM K^+^ and 2 mM Mg^+2^. Upon addition of 20 nM BLM in same salt conditions (in the absence of ATP), about 60% of initially folded GQ are unfolded as shown by the lower peak at E_FRET_ ≈0.19 (green histogram). B) The fraction of Cy3 spots that remain on PEG-Biotin surface is plotted as a function of time. Under identical conditions, about 10% of the spots are eliminated due to photobleaching (black rectangles) while more than 60% are eliminated when 20 nM BLM and 4 μM ATP are added. C) Cartoon showing how the unfolding of the GQ followed by unwinding of the duplex leads to the removal of the Cy3 strand from the surface, while Cy5 strand remains. The reduction in the Cy3 spots between t=0 and t=132 is clearly seen when the donor channels of the left image (t=0 s) and the middle image (t=132 s) are compared. In contrast, most of the initial Cy5 spots remained at t=132 s, as observed by exciting the same area with the red laser in the rightmost image.

**Supplementary References**

1. Janscak, P., Garcia, P.L., Hamburger, F., Makuta, Y., Shiraishi, K., Imai, Y., Ikeda, H. and Bickle, T.A. (2003) Characterization and mutational analysis of the RecQ core of the bloom syndrome protein. *J Mol Biol*, **330**, 29-42.

2. Budhathoki, J.B., Ray, S., Urban, V., Janscak, P., Yodh, J.G. and Balci, H. (2015) RecQ-core of BLM unfolds telomeric G-quadruplex in the absence of ATP. *Nucleic acids research*, **42**, 11528-11545.

3. Viglasky, V., Bauer, L., Tluckova, K. and Javorsky, P. (2010) Evaluation of human telomeric g-quadruplexes: the influence of overhanging sequences on quadruplex stability and folding. *Journal of nucleic acids*, **2010**.

4. Zhou, R., Zhang, J., Bochman, M.L., Zakian, V.A. and Ha, T. (2014) Periodic DNA patrolling underlies diverse functions of Pif1 on R-loops and G-rich DNA. *eLife*, **3**, e02190.

5. Gyimesi, M., Sarlos, K. and Kovacs, M. (2010) Processive translocation mechanism of the human Bloom's syndrome helicase along single-stranded DNA. *Nucleic acids research*, **38**, 4404-4414.

6. Chatterjee, S., Zagelbaum, J., Savitsky, P., Sturzenegger, A., Huttner, D., Janscak, P., Hickson, I.D., Gileadi, O. and Rothenberg, E. (2014) Mechanistic insight into the interaction of BLM helicase with intra-strand G-quadruplex structures. *Nature communications*, **5**, 5556.
